# Production of genetically engineered mice with higher efficiency, lower mosaicism, and multiplexing capability using maternally expressed Cas9

**DOI:** 10.1038/s41598-020-57996-7

**Published:** 2020-01-23

**Authors:** Takayuki Sakurai, Akiko Kamiyoshi, Hisaka Kawate, Satoshi Watanabe, Masahiro Sato, Takayuki Shindo

**Affiliations:** 1 Department of Life Innovation, Institute for Biomedical Sciences, Shinshu University, 3-1-1 Asahi, Matsumoto, Nagano 390-8621 Japan; 2 Department of Cardiovascular Research, School of Medicine, Shinshu University, 3-1-1 Asahi, Matsumoto, Nagano 390-8621 Japan; 3 Animal Genome Research Unit, Division of Animal Science, National Institute of Agrobiological Sciences, Ibaraki, 305-8602 Japan; 4 Section of Gene Expression Regulation, Frontier Science Research Center, Kagoshima University, 8-35-1 Sakuragaoka, Kagoshima, 890-8544 Japan

**Keywords:** Genetic engineering, CRISPR-Cas9 genome editing

## Abstract

The CRISPR/Cas9 system is widely used to generate gene-edited animals. Here, we developed an efficient system for generating genetically modified mice using maternal Cas9 from Cas9 transgenic mice. Using this system, we achieved lower mosaicism and higher rates of knock-in success, gene-editing, and birth compared to the similar parameters obtained using exogenously administered Cas9 (mRNA/protein) system. Furthermore, we successfully induced simultaneous mutations at multiple loci (a maximum of nine). Our novel gene-editing system based on maternal Cas9 could potentially facilitate the generation of mice with single and multiple gene modifications.

## Introduction

Genetically modified (GM) organisms, with specific genes altered (added or ablated), are widely used for modeling human and animal diseases. They are particularly useful for understanding the molecular mechanisms of diseases and for the development of novel disease treatments^1,2^. In the past three decades, GM animals have been created by microinjecting transgenes into zygote nuclei (zygote microinjection) or by injecting the blastocoel with GM embryonic stem cells engineered to exhibit altered expression of a specific gene by gene targeting technology^3,4^. It is now easier than ever to create GM animals by using the clustered regularly interspaced short palindromic repeat (CRISPR)/CRISPR-associated-9 (CRISPR/Cas9) genome editing system^5–12^ due to its wide applicability, high efficiency, and design simplicity^13–15^. The most commonly used approach for generating GM animals with the CRISPR/Cas9 system is by microinjecting the CRISPR/Cas9 components, such as Cas9 DNA, mRNA or protein, guide RNA (gRNA), and, in some cases, a homology direct repair (HDR) template, into the zygote^16–20^. gRNA efficiently induces Cas9-mediated double strand breaks at desired target sites, which stimulate DNA repair by at least two distinct mechanisms: non-homologous end joining and HDR^21–23^.

However, the zygote microinjection-based genome editing system has inherent problems. For example, frequent mosaicism for inducing insertion/deletion (indel) at the target locus is seen in almost all individuals obtained using this approach^24–26^. Given that transcription and translation are suppressed in mouse zygotes, the translation of the introduced Cas9 DNA/mRNA into its active enzymatic form is likely delayed until after the first cell division, causing unequal genome editing in individual blastomeres^27^. It has been recently reported that direct Cas9 protein expression in early-stage mouse zygotes reduces the occurrence of mosaic mutations^28^. In addition, the amount of CRISPR/Cas9 reagents injected into the zygote is limited as high volumes are often associated with developmental arrest of the embryos^29^, reducing the possibility of simultaneous modifications in multiple genes using the CRISPR/Cas9 system^11^. Previously, we tackled these problems by generating systemically Cas9-expressing transgenic (Tg) (sCAT) mice that produce maternal Cas9 (maCas9), which exhibits nuclease activity, during oogenesis^30^. Microinjecting gRNAs alone into the cytoplasm of sCAT zygotes resulted in the one-step generation of individuals carrying mutations in multiple genes, with gRNA decomposing around the 2-cell stage^30^.

One merit of utilizing maCas9 to generate GM animals is that they would exhibit less mosaicism for indel mutations because active Cas9 proteins would exist in the embryo only for a limited time. Furthermore, because the maCas9 approach does not require Cas9 mRNA (or protein) upon zygote microinjection, maCas9 zygotes may be injected with greater amounts of gRNA and, in some cases, with more DNA fragments. However, we found that gRNA microinjection into sCAT zygotes failed to reduce the mosaicism significantly^30^. We believe that this failure was probably due to the variable efficiencies of the gRNA preparations, which meant that the amounts of microinjected gRNAs differed among zygotes. This circumstance complicated the evaluation of maCas9-based genome editing. Furthermore, it has recently been reported that the delivery of CRISPR/Cas9 reagents into the zygote by electroporation (EP) provides higher genome editing efficiency than that achieved by zygote microinjection^31–33^.

In this study, we reevaluated the potential benefits of the maCas9-based genome editing system via the *in vitro* EP of sCAT zygotes with gRNAs and DNA fragments instead of zygote microinjection. First, we examined whether *in vitro* EP enabled the simultaneous and unbiased introduction of RNAs into zygotes and whether the efficiencies of inducing indel and knock-in (KI) mutations in maCas9 zygotes after *in vitro* EP with gRNA(s) alone were comparable to those in wild-type (WT) zygotes electroporated with gRNAs and *Cas9* mRNA (or protein). We also examined whether the indel introduction efficiency could be regulated by the concentration of gRNA introduced into sCAT zygotes and whether there were fewer mosaic mutations in sCAT zygotes electroporated with gRNA alone than in WT zygotes electroporated with *Cas9* mRNA and gRNA. Finally, we examined whether multiple (up to 10) genes can be knocked out simultaneously in sCAT zygotes electroporated with multiple gRNAs. This study shows that maCas9-based gene-editing is a potential alternative system for generating animals with various single and multiple gene modifications.

## Results

### *In vitro* EP prompts synthetic mRNA uptake by zygotes with little variation between individuals

We first examined whether the *in vitro* EP of zygotes in the presence of an external substance (i.e., synthetic mRNA, such as *EGFP A95* mRNA) permits incorporation of the latter into zygote cytoplasm and assessed the extent of variation in incorporation efficiency between individuals. We prepared 5 µL aliquots of EP solution containing 0, 20, 100, or 200 ng/mL *EGFP A95* mRNA and placed them between two electrodes on a plate. Zygotes derived from *in vitro* fertilization (IVF; 8–10 embryos/group) were then introduced into the drop and immediately subjected to *in vitro* EP. These experiments were carried out twice. The EP-treated zygotes were then allowed to develop into 2-cell embryos after being cultured for 12 h *in vitro*. In these developing 2-cell embryos, the average *EGFP A95*-derived fluorescence intensity, measured using a fluorescence microscope, did not vary among embryos of different groups (Fig. S1a), and the fluorescence intensity was directly proportional to the amount of *EGFP A95* mRNA used (Fig. S1b). In contrast, variable fluorescence intensities were observed among embryos when the zygotes were microinjected with 20 ng/μL *EGFP A95* mRNA (Fig. S1c). These results indicate a close relationship between the amount of mRNA incorporated into zygotes and the rate at which the mRNA is translated into proteins in the resultant embryos. Next, we used *in vivo* EP to examine the possible utility of maCas9 for improving the overall efficiency of genome editing using sCAT-derived zygotes.

### Regulation of indel induction efficiency by varying concentration of gRNA introduced into sCAT zygotes

**Figure 1:**
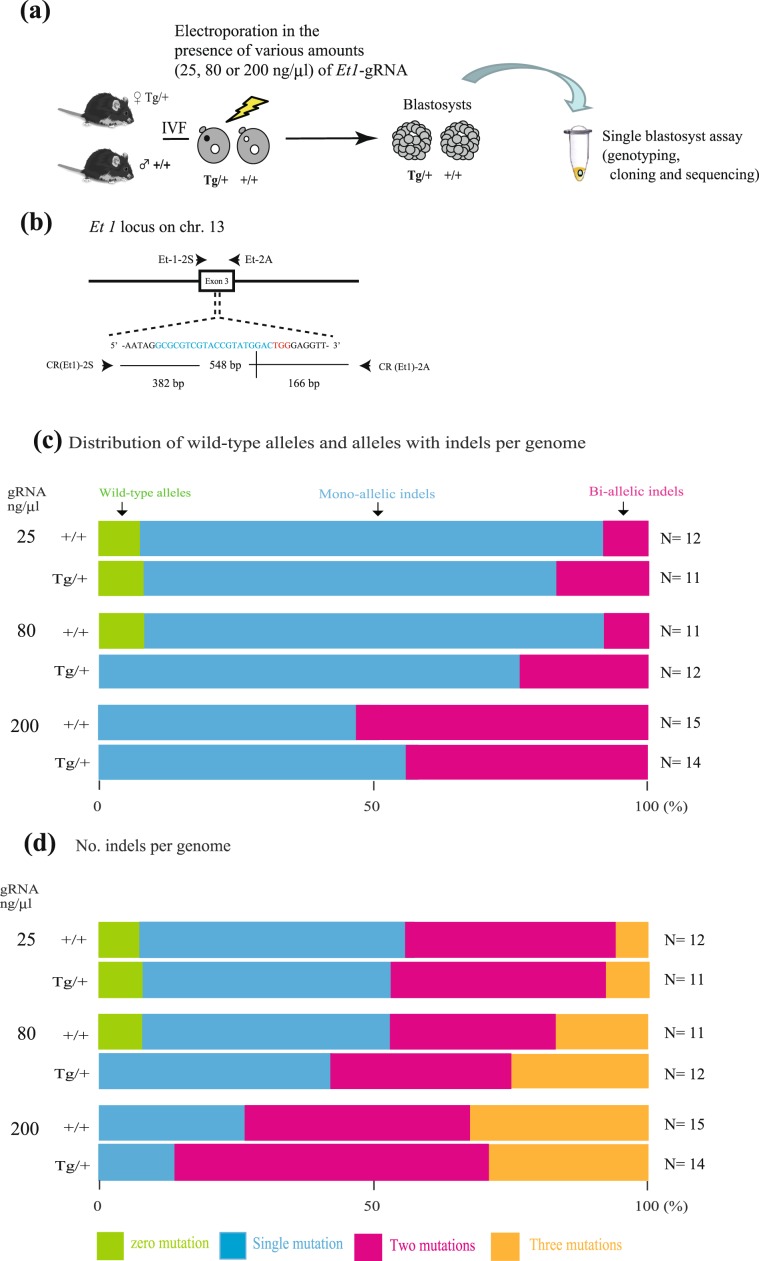
Relationship between gRNA concentration and the number of induced indels. (**a**) Schematic of the experimental procedure for examining the number of indels induced in maCas9 zygotes after electroporation with various amounts of *Et1*-gRNA. (**b**) *Et1*-gRNA targeting sites in exon 3 of the murine *Et1* gene. The *Et1*-gRNA-coding sequence is shown in blue and the protospacer adjacent motif sequence is shown in red. Arrows indicate the locations of the PCR primers (see Table S5). (**c**) Distribution of wild-type alleles and alleles with indels per genome in blastocysts developed from zygotes electroporated with various amounts of *Et1*-gRNA. (**d**) Number of indels per genome in blastocysts developed from zygotes electroporated with various amounts of *Et1*-gRNA.

Maximal accumulation of maCas9 in oocytes has been hypothesized to occur during folliculogenesis^27^. Thus, the amount of maCas9 in each ovulated oocyte should be sufficient for genome editing and individual differences should be quite small, in which case, it may be possible to regulate the efficiency of indel generation by changing the amount of gRNA introduced into the oocytes. Therefore, we examined the relationship between gRNA concentration and the degree of indel generation using the experimental procedure outlined in Fig. 1a: sCAT-derived zygotes were subjected to *in vitro* EP in the presence of 25, 80, or 200 ng/µL gRNA targeting the endothelin1 (*Et1*) gene (hereafter referred to as *Et1*-gRNA), and after EP, the treated zygotes were allowed to develop into blastocysts *in vitro*. The blastocysts were lysed to isolate genomic DNA for the PCR-based amplification of the target region recognized by *Et1-*gRNA (Figs. 1b and S2a). The resulting PCR products were then cloned into the TA cloning vector for sequencing; typical examples are shown in Fig. S2b. When *Et1*-gRNA was introduced at a concentration of 25 ng/µL (Fig. 1c), 8% of +/+ blastocysts and 9% of Tg/+ blastocysts had two WT alleles, whereas 8% and 18% of +/+ blastocysts and Tg/+ blastocysts had indels in both alleles (hereafter referred to as “bi-allelic” mutations), respectively. Moreover, 84% of the +/+ blastocysts and 73% of the Tg/+ blastocysts carried indels in only one of the two alleles (hereafter referred to as “mono-allelic” mutations), and there was no significant difference between the percentage of +/+ and Tg/+ blastocysts with indels per genome. Similar results were obtained when *Et1*-gRNA was introduced at concentrations of 80 and 200 ng/µL. However, in the latter case, there were no +/+ or Tg/+ blastocysts carrying WT alleles and the fraction carrying mono-allelic mutations decreased to 47–57%, whereas the fraction carrying bi-allelic mutations increased to 47–57% (Fig. 1c). The efficiency of indel induction per genome positively correlated with the amount of gRNA introduced by *in vitro* EP (Fig. 1d), and there was no significant difference in the fraction of +/+ and Tg/+ blastocysts with indels per genome, suggesting that it may be possible to regulate the rate of indel induction via the concentration of gRNA introduced into zygotes carrying maCas9. We assessed this hypothesis by producing *Et1* gene-modified mice (Fig. S3). Briefly, sCAT-derived zygotes were subjected to *in vitro* EP with 25 or 200 ng/µL *Et1*-gRNA (Fig. S3a) and transferred into the oviducts of pseudopregnant recipient females one day after cultivation to obtain pups (Fig. S3b). Phenotypic alterations in the pups (e.g., craniofacial defects) and the presence of possible mutations in the target sequence recognized by *Et1*-gRNA were assessed (Fig. S3c). As expected, no *Et1* knockout (KO) pups with craniofacial defects were obtained when EP was carried out with 25 ng/µL *Et1*-gRNA, with the efficiency of indel induction ranging between 57 and 67%. In contrast, when EP was carried out with 200 ng/µL *Et1*-gRNA, 29–56% of pups were identified as *Et1* KO, and all of them carried indels.

### Highly efficient genome editing in maCas9 zygotes electroporated with 200 ng/µL gRNA

**Figure 2:**
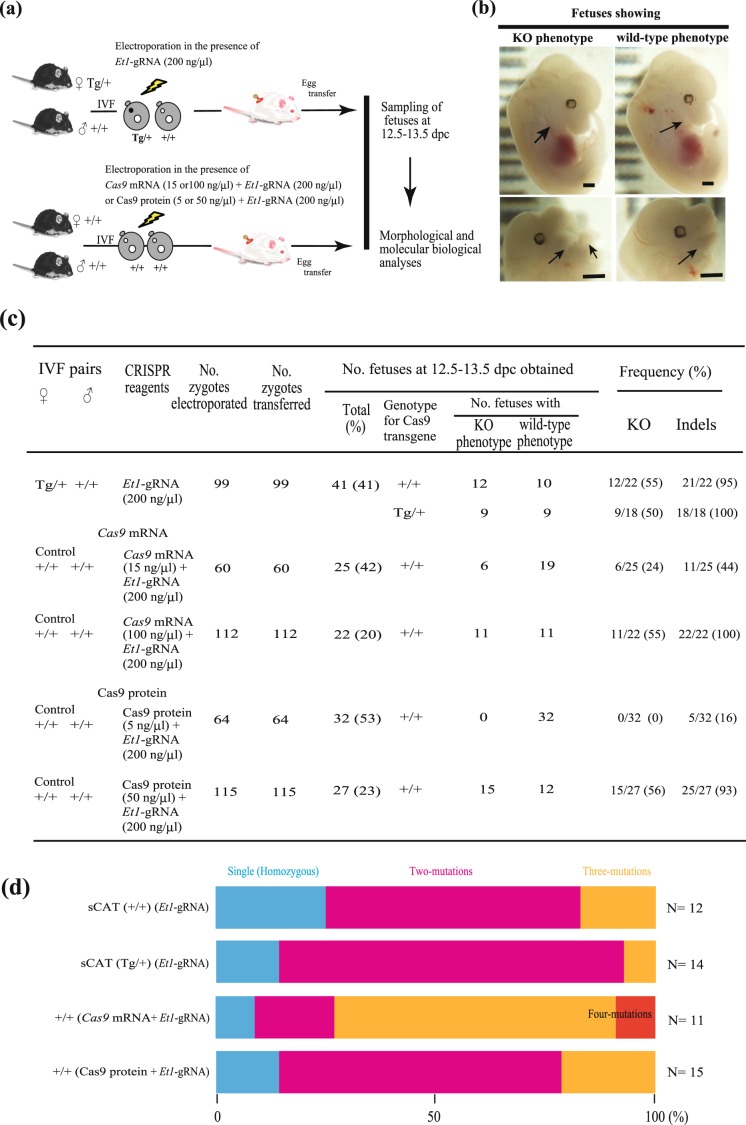
Comparison of genome editing efficiency in maCas9 and wild-type (WT) zygotes. (**a**) Schematic of the experimental procedure for examining the efficiency of *Et1* gene-editing in maCas9 and WT (non-maCas9) zygotes. (**b**) Representative 13.5-day post coitum fetuses exhibiting *Et1* knockout (KO) and WT phenotypes. Arrows indicate (left panel; KO phenotype) morphological abnormalities of the craniofacial tissue derived from the pharyngeal arch and (right panel; WT phenotype) normal craniofacial tissue. Scale bars indicate 1 mm. (**c**) Table summarizing the efficiency of *Et1* gene-editing observed in maCas9 and WT zygotes after the transfection with exogenous Cas9 mRNA (or protein) and gRNA. (**d**) Number of indels per genome in *Et1* KO fetuses developed from maCas9 zygotes (+/+ and Tg/+) electroporated with 200 ng/µL *Et1*-gRNAs and non-maCas9 zygotes electroporated with 200 ng/µL *Et1*-gRNAs and various amounts of exogenous Cas9 mRNA (or protein).

We compared the efficiency of genome editing in maCas9 zygotes and non-maCas9 zygotes co-administered with *Cas9* mRNA/gRNA or Cas9 protein/gRNA (Fig. 2a). First, maCas9 zygotes were electroporated in the presence of 200 ng/µL *Et1-*gRNA and transferred to recipient females to develop. On days 12.5–13.5, fetuses with craniofacial defects (Fig. 2b) were deemed to be *Et1* KO fetuses, as described in a previous gene targeting-based study^34^. All fetuses were subjected to genotyping for the presence of the Cas9 transgene, PCR of the target region recognized by *Et1*-gRNA, and sequencing of the PCR products cloned into a TA cloning vector. Typical results obtained from this experiment are shown in Fig. S4. Control non-maCas9 zygotes derived from the IVF of oocytes (+/+) with sCAT spermatozoa (+/+) were subjected to *in vitro* EP with 15 ng/µL *Cas9* mRNA/200 ng/µL *Et1* gRNA, 100 ng/µL *Cas9* mRNA/200 ng/µL *Et1*-gRNA, 5 ng/µL Cas9 protein/200 ng/µL *Et1-*gRNA, or 50 ng/µL Cas9 protein/200 ng/µL *Et1-*gRNA (Fig. 2a). The treated embryos were then transferred into recipient females to obtain mid-gestational fetuses, and molecular biological analysis was performed as described above (Fig. 2c). Of the fetuses derived from maCas9 zygotes (Fig. 2a, upper panel), 55% of the +/+ fetuses and 50% of the Tg /+ fetuses had *Et1* KO phenotype, whereas nearly 100% had indels, as expected (Figs. 1c and S3c). These results appear to correspond to those obtained from the non-maCas9 zygotes electroporated with 100 ng/µL *Cas9* mRNA/200 ng/µL *Et1*-gRNA or 50 ng/µL Cas9 protein/200 ng/µL *Et1-*gRNA (Fig. 2c), since the rates of KO incidence in these groups were both high and not significantly different. Next, we checked for possible off-target effects induced by *Et1*-gRNA in *Et1* KO fetuses (12 +/+ fetuses and 9 Tg/+ fetuses). No mutations in the target regions listed as those with higher probability have been observed (Table S1). We also compared the efficiency of genome editing in maCas9 and non-maCas9 zygotes after co-administering *Cas9* mRNA/gRNA or Cas9 protein/gRNA, both of which target *Tyr* (Fig. S5a). The incidence rates of *Tyr* KO phenotype (evaluated by eye pigment deficiency; Fig. S5b) and indel induction were relatively high and not significantly different (Fig. S5c) among the three groups (47–68% and 75–84%, respectively).

### Low mosaic mutation rate in sCAT zygotes electroporated with 200 ng/µL gRNA

To examine mosaic mutation efficiency in maCas9 zygotes, we compared the distribution of indel modifications in mid-gestational fetuses derived from maCas9 zygotes electroporated with 200 ng/µL gRNA and from non-maCas9 zygotes electroporated with *Cas9* mRNA (100 ng/µL)/gRNA (200 ng/µL) or Cas9 protein (50 ng/µL)/gRNA (200 ng/µL) (Fig. 2d). *Et1* KO fetuses (9–15 per group) were selected from each group (Fig. 2c). For the Tg/+ samples, the nine fetuses shown in Fig. 2c and five fetuses shown in Fig. S3 were combined and analyzed (Fig. 2d). The percentages of maCas9 zygote-derived fetuses (+/+) with one, two, or three indels after EP with 200 ng/µL *Et1-*gRNA were 25, 58, and 17%, respectively. Similarly, the percentages of Tg/+ fetuses with one, two, or three indels were 14, 79 and 7%, respectively (Fig. 2d), whereas the percentages of Tg/+ fetuses with one, two, or three/four indels after EP with *Cas9* mRNA/*Et1*-gRNA were 9, 18, and 73% respectively (Fig. 2d). Moreover, the percentages of fetuses with one, two, or three indels after EP with *Cas9* protein/Et1-gRNA were 13, 67, and 20%, respectively (Fig. 2d). There were no significant differences in the percentages of indel generation between sCAT +/+ (*Et1*-gRNA), Tg/+ (*Et1*-gRNA), and +/+ (*Cas9* protein/*Et1*-gRNA) groups (Fig. 2d). However, significant differences were observed in indel percentages in sCAT +/+ (*Et1*-gRNA), sCAT Tg/+ (*Et1*-gRNA), and +/+ (*Cas9* protein/Et1-gRNA) vs. +/+ (*Cas9* mRNA/*Et1*-gRNA) samples (*P* = 0.023, 0.001, and 0.042, respectively; Fig. 2d). These results indicate that the mosaic mutation rate of maCas9 zygotes treated with *Et1*-gRNA alone is lower than that of non-maCas9 zygotes treated with *Cas9* mRNA/*Et1*-gRNA and equivalent to that of non-maCas9 zygotes treated with Cas9 protein/*Et1*-gRNA (Fig. 2d). Similar results were obtained when another *Tyr* was examined (Fig. S5d; sCAT (*Tyr*-gRNA) vs. +/+ (*Cas9* mRNA/*Tyr* -gRNA); *P* = 0.037). Furthermore, these results were also confirmed using indel analysis by Inference of CRISPR Edits 2 software (ICE2) (Fig. S6). The rate (%) of mosaicism (presence of three indels or more) of sCAT Tg/+ and +/+ (*Tyr*-gRNA) was 34%, which was lower than that of +/+ (*Cas9* mRNA/*Tyr*-gRNA) (58%) or +/+ (*Cas9* protein/*Tyr*-gRNA) (50%). Thus, the rate of mosaic mutations in maCas9 zygotes (introduced with gRNA alone) may be lower than in non-maCas9 zygotes (introduced with *Cas9* mRNA/gRNA).

### Similar KI mutation efficiency in maCas9 zygotes and non-maCas9 zygotes transfected with Cas9 mRNA/gRNA or Cas9 protein/gRNA by electroporation

**Figure 3:**
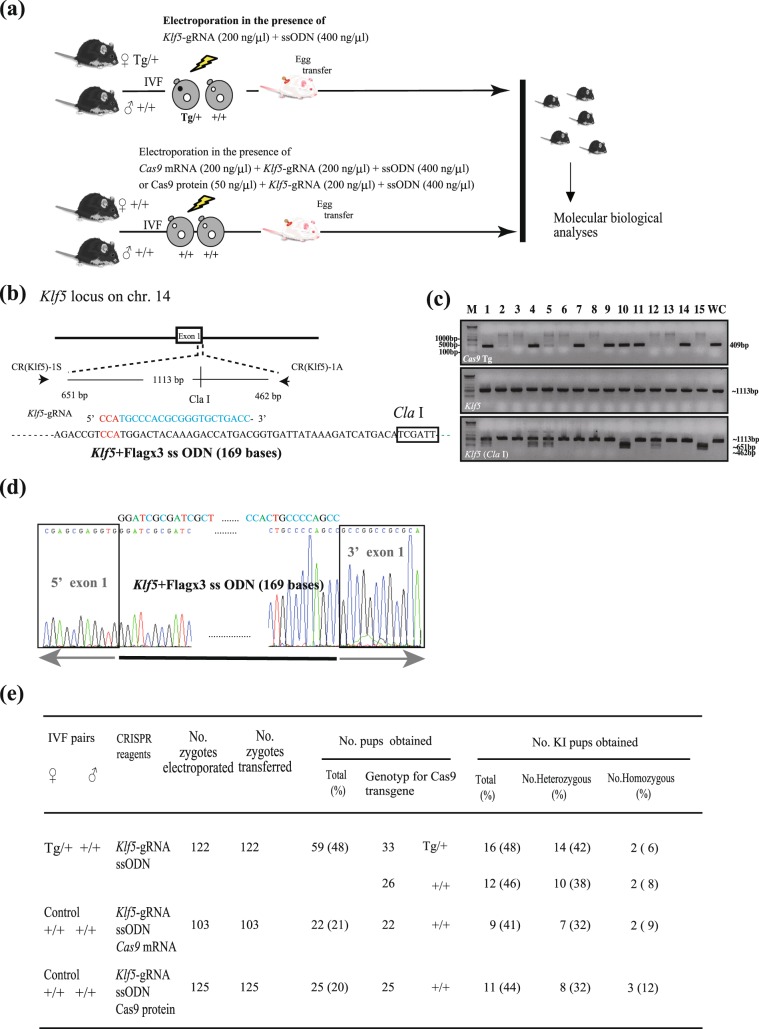
Comparison of knock-in (KI) mutation efficiency in maCas9 and wild-type (WT) zygotes. (**a**) Schematic of the experimental procedure for examining KI mutation efficiency using *Klf5* + *flag* × *3* ssODN targeting the *Klf5* gene. (**b**) *Klf5*-gRNA targeting exon 1 of the murine *Klf5* gene. The sequence recognized by *Klf5*-gRNA is shown in blue and the protospacer adjacent motif sequence is shown in red. Arrows indicate the locations of the PCR primers (see Table S5). All *Klf5* + *flag* × *3* ssODN sequences are shown in Table S5. (**c**) Representative image of agarose gel electrophoresis of PCR products amplified from the genomic DNA of pups (nos. 1–15) obtained from maCas9 zygotes electroporated with 200 ng/µL *Klf51*-gRNAs and 400 ng/µL ssODN. Top panel: genotyping for the Cas9 gene. Middle and bottom panels: results of the restriction fragment length polymorphism assay using the *Cla* I enzyme. Pups 1, 4, 5, and 12 were heterozygous for the KI mutation. Pups 10 and 15 were homozygous for the KI mutation. Lanes 1–15 show PCR products amplified from the genomic DNA of all pups; lane WC, PCR product amplified from the genomic DNA of a WT pup; M, lambda *Hin* dIII + 100-bp ladder markers. Full-length gel images are presented in Supplementary Information Fig. 3(c). (**d**) Sequences of 5′ and 3′ junction sites in pup 10. Sequence chromatograms show correct KI of *Klf5* + *flag* × *3* ssODN (Table S5) into exon 1 of the *Klf5* gene. (**e**) Table comparing KI mutation induction efficiency of maCas9-based genome editing and genome editing in WT zygotes after transfection with exogenous Cas9 mRNA (or protein) and gRNA.

Next, we examined KI mutation efficiency in maCas9 zygotes using gRNA targeted to the *Klf5* gene (hereafter referred to as *Klf5-*gRNA; Fig. 3a,b). maCas9 zygotes were subjected to *in vitro* EP with 200 ng/µL *Klf5*-gRNA and 400 ng/µL single-stranded oligodeoxynucleotide (ssODN). Control non-maCas9 zygotes were electroporated with 200 ng/µL *Cas9* mRNA, 200 ng/µL *Klf5*-gRNA, and 400 ng/µL ssODN or 50 ng/µL Cas9 protein, 200 ng/µL *Klf5*-gRNA, and 400 ng/µL ssODN. Treated embryos were transferred into the oviducts of pseudopregnant females to obtain pups, whose tails were used to extract genomic DNA, which was subjected to genotyping to detect Cas9 transgenes, PCR of the target sequence recognized by *Klf5*-gRNA, and the restriction fragment length polymorphism assay using the *Cla* I enzyme. Typical results are shown in Fig. 3c (e.g., #1, #4, and #10). PCR products, obtained using genomic DNA from randomly selected *Cla* I site-positive mice as PCR template, were sequenced to examine whether ssODNs were correctly knocked-in at the target locus (Fig. 3d). The efficiency of KI mutation in maCas9 zygotes was 48% for Tg/+ pups and 46% for +/+ pups (Fig. 3e), with no significant difference. Furthermore, the efficiency of KI mutation generated by using non-maCas9 zygotes into which *Cas9* mRNA/gRNA/ssODN or Cas9 protein/gRNA/ssODN had been introduced was slightly lower, 41–44%, which did not show statistical significance (Fig. 3e).

We also examined the generation of KI mutations in maCas9 zygotes by using gRNA targeted to the androgen receptor gene (hereafter referred to as *Ar*-gRNA; Fig. S7a,b) based on the report of Shen *et al*.^35^, who showed that *Ar*-gRNA has off-target activity towards at least two genomic loci (3140158–3140172 on chromosome 11 and 121017073–121017287 on chromosome 8; Table S2). To assess the possible off-target activity at these two loci, maCas9 zygotes were subjected to *in vitro* EP with 100 ng/µL *Ar*-gRNA and 200 ng/µL ssODN. Control non-maCas9 zygotes were electroporated with 100 ng/µL *Cas9* mRNA, 100 ng/µL *Ar*-gRNA, and 200 ng/µL ssODN or 20 ng/µL Cas9 protein, 100 ng/µL *Ar*-gRNA, and 200 ng/µL ssODN. The treated zygotes were allowed to develop into blastocysts *in vitro* (Fig. S7a) and then lysed to isolate genomic DNA for PCR-based amplification of the target sequence recognized by *Ar-*gRNA (Fig. S7b,c) and RFLP assays using *Bam* HI enzyme. Typical KI mutations are shown in Fig. S7c (e.g., #2 and #8). The sequencing results of the PCR products (#8) are shown in Fig. S7d. Blastocysts derived from maCas9 zygotes exhibited KI mutation rate of 8% (Fig. S7e) and off-target activity on chromosomes 8 (genome position 121017073–121017287) and 11 (genome position 3140158–3140172) at mutation rates of 7.9% and 2.8%, respectively (Table S2).

### maCas9 allows efficient simultaneous modification of multiple genes

**Figure 4:**
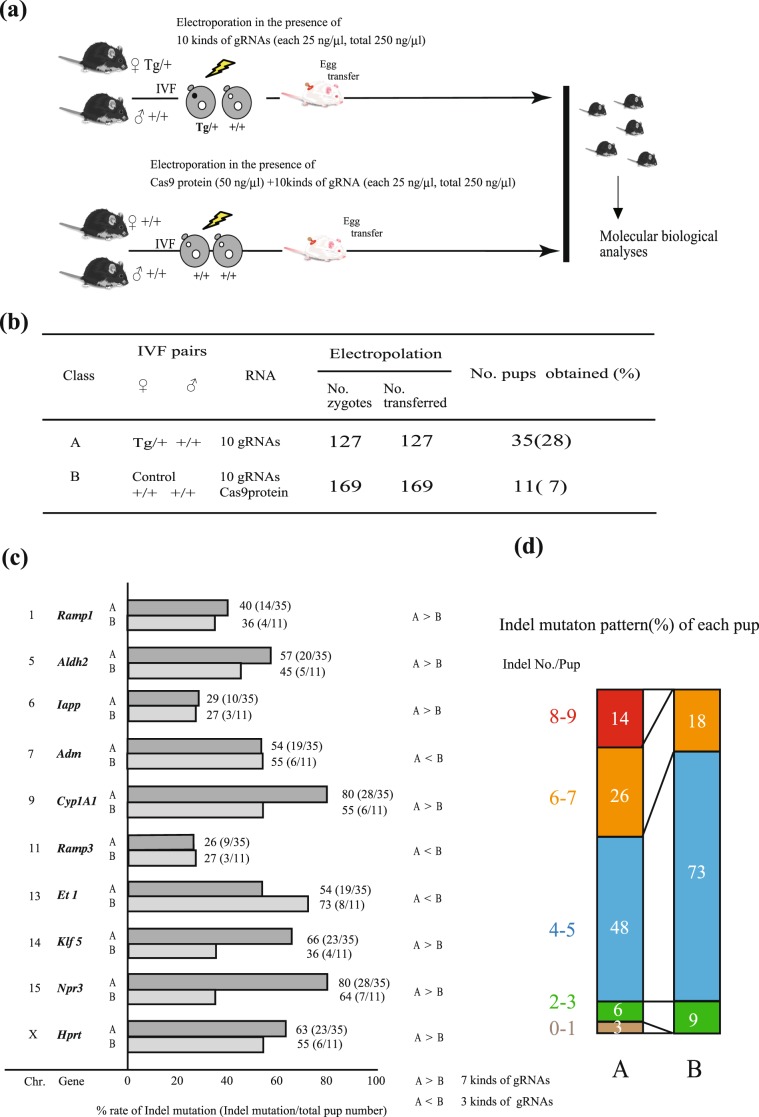
Superiority of maCas9-based genome editing for simultaneously modifying multiple genes in zygotes. (**a**) Schematic of the experimental procedure for examining the efficiency of simultaneously modifying multiple (10) target loci by genome editing in maCas9 zygotes. The sequences of the 10 target genes are listed in Fig. 4c and Table S4. (**b**) Table comparing the birth rates of pups developed from maCas9 zygotes electroporated with 10 gRNAs (25 ng/µL each; class A) or non-maCas9 wild-type zygotes electroporated with 10 gRNAs (25 ng/µL each) and 50 ng/µL exogenous Cas9 protein (class B). The Cas9 protein (50 ng/µL) concentration was chosen based on the results presented in Fig. 2c. (**c**) Indel frequencies for each of the 10 genes in the pups shown in (**b**). Chr., chromosome number. A > B and A < B indicate whether the indel frequency in class A was higher or lower than that in class B, respectively. (**d**) Distribution of different indel combinations in class A and class B pups. Values on the graph indicate the percentages of pups with the respective number of simultaneously induced indels.

We next explored the possibility of using maCas9-based genome editing to simultaneously modify multiple genes. maCas9 zygotes were electroporated with 10 gRNAs for *Adm, Amy, Aldh2, Cyp1a1, Et1, Hprt, Klf5, Npr*3*, Ramp1*, and *Ramp*3 (Table S4) at a concentration of 25 ng/µL for each gene (Fig. 4a). Control non-maCas9 zygotes were electroporated in a solution containing the 10 gRNAs mentioned above at concentrations of 25 ng/µL and 50 ng/µL Cas9 protein. The electroporated zygotes were transferred into recipient females to produce pups, with birth rates of 28% (35/127) and 7% (11/169) in the experimental and control groups, respectively (Fig. 4b). The indel mutation efficiency of each target gene per individual ranged from 26 to 80%, which may reflect the different abilities of the gRNAs to achieve optimal genome editing efficiency. However, the maCas9 zygotes (experimental group) exhibited higher genome editing efficiencies than the non-maCas9 zygotes (WT control group) at seven of the ten loci examined (Fig. 4c). Notably, the number of mice carrying mutations at 8–9 loci was significantly higher for the transferred maCas9 zygotes than the transferred non-maCas9 zygotes (Fig. 4d; *P* = 0.007), as was the number of mice carrying mutations in 6–7 loci (Fig. 4d; *P* = 0.0132). To assess possible off-target activities, we examined three possible off-target loci in each of the five genes, *Cyp1A1, Et1, Klf5, Npr*3*, and Hprt*, all of which exhibited > 50% on-target activity (Fig. 4c) with no observable mutations in the target regions (Table S3).

Based on these data, we generated GM mice in which three different loci (*Et1, Ramp1*, and *Ramp3*) had been simultaneously disrupted (Fig. S8). maCas9 zygotes were electroporated with 25 ng/µL *Et1* gRNA, 100 ng/µL gRNA targeted to *Ramp1* (hereafter referred to as *R1-*gRNA), and 100 ng/µL gRNA targeted to *Ramp3* (hereafter referred to as *R3-*gRNA; Fig. S8a). The *Et1* gRNA concentration was lowered to 25 ng/µL to avoid possible embryonic lethality caused by a complete loss of *Et1* expression^34^, based on the experiments shown in Figs. 1 and S3. The electroporated zygotes were transferred to pseudopregnant females to produce pups, with a birth rate of 43% (37/86; Fig. S8b). Newborn mice with Tg/+ or +/+ genotypes exhibited high average indel rates for each locus (57% for *Et1*, 73% for *Ramp1*, and 59% for *Ramp3*; Fig. S8b). Genomic DNA was isolated from these pups and genotyped for the presence of Cas9 transgenes. Fig. S8c shows the results of PCR-based amplification of target sequences recognized by each gRNA and a Cas9 ribonucleoprotein (RNP) cut assay for identifying bi-allelic indel mutations. Typical results are shown in Fig. S9. Of the 15 mice tested, 20% (3/15) had indels at three loci, 53% (8/15) at two loci, and 20% (3/15) at one locus. The genomes of the remaining mice (7%, 1/15) were unedited.

### maCas9 zygotes generate more genome-edited pups than WT zygotes co-transfected with *Cas9* mRNA/gRNA or Cas9 protein/gRNA

Notably, maCas9 zygotes delivered a larger number of genome-edited pups than non-maCas9 WT zygotes co-transfected with *Cas9* mRNA/gRNA or Cas9 protein/gRNA. For example, maCas9 zygotes electroporated with *Et1* gRNA generated more pups than WT zygotes co-transfected with *Cas9* mRNA/gRNA (41% *vs*. 20%, *P* = 0.014; Fig. 2c) or Cas9 protein/gRNA (41% *vs*. 23%, *P* = 0.049; Fig. 2c). A total of 58% of the maCas9 zygotes electroporated with *Tyr*-gRNA developed into newborn pups, which is higher than the percentage of WT zygotes co-transfected with Cas9 mRNA/gRNA or with Cas9 protein/gRNA (45% and 32%, respectively; Fig. S5c); however, there was no significant difference between these groups. Similarly, in the case of KI mutation induction at the *Klf5* locus, maCas9 zygotes generated more newborn pups than non-maCas9 zygotes electroporated with *Cas9* mRNA/gRNA (48% *vs*. 21%, *P* = 0.004; Fig. 3e) or Cas9 protein/gRNA (48% *vs*. 20%, *P* = 0.001; Fig. 3e). When KI mutations were induced at the *Ar* locus, maCas9 zygotes developed more blastocysts than non-maCas9 zygotes electroporated with *Cas9* mRNA/gRNA (69% *vs*. 15%, *P* = 0.0002; Fig. S7e) or Cas9 protein/gRNA (69% *vs*. 8%, *P* = 0.0001; Fig. S7e). Finally, when 10 gRNAs were introduced simultaneously, maCas9 zygotes developed into viable pups at a greater rate than WT zygotes electroporated with Cas9 protein/gRNAs (28% *vs*. 11%, *P* = 0.0001; Fig. 4b).

## Discussion

The maCas9-mediated generation of genome-edited mice, first reported by us^30^, can be performed by introducing gRNA alone into maCas9-containing eggs without the need to co-introduce exogenous Cas9 mRNA (or protein). This technology enables multiple target loci in the genome to be edited simultaneously. In this study, we elaborated our previous method by examining the properties of this technology in more detail. In particular, we compared this approach with the most widely used method for the *in vitro* EP-mediated generation of genome-edited mice, which uses a combination of gRNA and Cas9 mRNA (or protein). We found that our system exhibits a comparably high degree of indel mutation efficiency and is superior to the other system due to a low degree of mosaicism, higher KI mutation efficiency, increased pup delivery rate, and the ability to induce mutations in multiple loci simultaneously.

Since we first reported this method^30^, similar studies have been published by other groups. Zhang *et al*. generated Tg mice in which Cas9 expression was limited to oocytes during oogenesis under the transcriptional control of the oocyte-specific promoter ZP3^36^. Eggs derived from this Tg line were associated with a lower frequency of mosaicism and a lower occurrence of off-target mutations compared to those observed with conventional methods in which gRNA and *Cas9* mRNA are introduced into normal fertilized eggs. However, the system designed by Zhang *et al*.^36^ failed to induce KO mutations at a high rate. The authors speculated that this failure was likely because the amounts of maCas9 in the immature oocytes were too low. Furthermore, Cebrian-Serrano *et al*. generated a Tg line carrying a transgene composed of the chicken β-actin-based promoter (CAG), *Cas9* gene, and poly(A) site inserted into the *Gt(ROSA26)Sor* locus^37^. Using eggs derived from this line, they demonstrated that maCas9-based genome editing induced indels at a level comparable to that achieved with the conventional methods based on the introduction of gRNA and exogenous Cas9 mRNA (or protein) and achieved higher KI mutation efficiency in the target insert. These two reports are generally consistent with our data; however, our results were slightly differently. This discrepancy may have been caused by the use of different gRNAs or promoters to drive Cas9 gene expression, different chromosomal locations of the integrated transgenes, and different genetic backgrounds of the mice, all of which may affect the maCas9 accumulation rate in oocytes. It is difficult to compare the ability to induce genome editing in the target genes of the above-mentioned Cas9-expressing Tg mice. Therefore, in this study we compared the overall genome-edited mouse generation efficiency of our maCas9-based genome editing system using maCas9-expressing Tg mice and the widely used genome editing system based on the introduction of gRNA and Cas9 mRNA (or protein) in-house.

To do so, we used *in vitro* EP-based gene delivery to introduce genome editing components into fertilized eggs as it is easy to handle, does not require a high degree of manipulator skill, and allows several embryos to be treated at the same time^31–33^. Having compared the efficiencies of the zygote microinjection method, which is also widely used to generate genome-edited mice, and *in vitro* EP-mediated gene transfer, we examined whether *EGFP A95* mRNA could be introduced into fertilized eggs in a non-biased fashion. The latter technique was found to be better because: 1) embryonic fluorescence intensity did not vary between the groups; and 2) fluorescence intensity was directly proportional to the amount of *EGFP A95* mRNA used (Fig. S1). Given that the *in vitro* EP-based method allows unbiased introduction of a relatively constant amount of gRNA and Cas9 mRNA (or protein) into embryos and is suitable for quantitative analysis, we used this method of gene transfer in subsequent experiments.

Notably, there was a relationship between the amount of gRNA introduced into maCas9-containing eggs and the number of indels generated per genome (Fig. 1), meaning that the amount of maCas9 was almost equal in each egg. Furthermore, it may be possible to preferentially generate F0 mice with mono-allelic mutations (and bi-allelic mutations) at target loci by controlling the amount of gRNAs used. This could be particularly useful when researchers need to edit a mouse gene whose complete disruption causes embryonic lethality. Indeed, in our study we demonstrated that *in vivo* EP with maCas9 and a low concentration of gRNA (25 ng/µL) targeting *Et1*, a gene whose complete KO is known to cause embryonic lethality^34^, preferentially generated pups with mono-allelic mutations (Figs. 1c, S3, and S8).

To evaluate the exact genome editing ability of maCas9 in oocytes, we must consider whether oocytes have Cas9 transgenes or not (so-called Tg/+ oocytes or +/+ oocytes). For CAG promoter-containing transgenes, Cas9 transcription and translation in eggs carrying the transgenes occur from the 4-cell stage onward^38^, often yielding misleading results when maCs9-mediated genome editing activity is evaluated. Zhang *et al*.^36^ eliminated the possibility that the Cas9 protein is synthesized from transgenes in their Tg lines during the cleavage stage of embryonic development by using the ZP3 promoter to drive Cas9 expression, which is inactive at the cleavage stage. Cebrian-Serrano *et al*.^37^ did not distinguish Tg/+ eggs from +/+ eggs when evaluating genome editing efficiency in their Tg lines. In our study, genome editing efficiency of maCas9-containing eggs was not affected by the presence of transgenes (Figs. 1c,d, 2c, and 3e), suggesting that: (1) maCas9, added as a maternal factor during oogenesis, might have been degraded like other general maternal mRNA/proteins before the 2-cell stage^27,39^; and (2) almost all gRNAs introduced into the zygotes might have been inactivated (and thus degraded) by the time the zygotic genes are activated and *de novo* Cas9 proteins are translated. Therefore, in subsequent experiments, we analyzed a mixture of +/+ and Tg/+ maCas9 zygotes. We also performed immunostaining, but failed to detect maCas9 protein in ovulated oocytes to 8-cell embryos (obtained from sCAT mice), despite using several approaches: DAB-based staining, Alexa 488-based fluorescent staining, the use of different fixation (paraformaldehyde or ethanol), permeabilization (Tween 20 or Triton X100), and blocking (bovine serum albumin or serum) solutions, as well as various commercial available Cas9 antibodies (Diagenode Inc., Belgium, C15200216; TaKaRa BIO Inc., Shiga, Japan, Z2607N) and anti-FLAG tag antibodies (Sigma, F-3165; Transgenics Inc., Kobe, Japan, KO602-S).

In this study, we successfully demonstrated that maCas9-based genome editing efficiently induces KO indels in 55% of +/+ individuals and 50% of Tg/+ individuals (Fig. 2), comparable to the genome editing efficiency following the introduction of exogenous Cas9 mRNA (or protein) and gRNA to obtain *Et1* KO offspring. In this case, maximum indel induction (as high as 56%) was achieved when exogenous Cas9 protein and gRNA were introduced into WT eggs (Fig. 2), and similar results were obtained when targeting the *Tyr* gene (Figs. S5 and S6). These results suggest that the enzymatic activity of the exogenously introduced active Cas9 protein (at 50 ng/µL) is slightly higher than that of maCas9 in sCAT eggs. A similar observation was also made by Zhang *et al*. (2016), who used eggs derived from the ZP3-Cas9 Tg line for maCas9-based genome editing^36^. As for the induction of mosaic mutations, our maCas9-based genome editing system caused low mosaicism in *Et1* KO offspring and *Tyr* KO offspring, similar to that observed in the case of a conventional genome editing system with RNP (comprising Cas9 protein and gRNA; Figs. 2d, S5d, and S6b) and consistent with the results of Zhang *et al*.^36^ and Cebrian-Serrano *et al*.^37^. In contrast, the *in vitro* EP of WT eggs in the presence of exogenous *Cas9* mRNA generated higher ratios of mosaic offspring (see Figs. 2d, S5d, and S6b), potentially due to a delay in the translation or action of the Cas9 protein after introducing *Cas9* mRNA into the embryo^27,39^. The efficiency of KI mutation by the maCas9-based genome editing system using ssODN and gRNA targeting *Klf5* tended to be higher than that of genome editing systems based on the introduction of *Cas9* mRNA or protein (48% *vs*. 41% and 44%, respectively; Fig. 3). We also confirmed that KI mutations in the *Ar* gene were induced in the blastocysts derived from maCas9 zygotes (Fig. S7). Notably, Cebrian-Serrano *et al*.^37^ also demonstrated that their maCas9-based genome editing system had a slightly higher KI mutation efficiency (36% *vs*. 19–30%) than genome editing systems based on the introduction of exogenous Cas9 mRNA (or protein) and gRNA. CRISPR/Cas9-mediated KI events are thought to occur via HDR-mediated gene replacement or insertion^40^, which are probably not affected by Cas9 molecules enriched in fertilized eggs.

Furthermore, we confirmed that the maCas9-based genome editing system is more efficient than the conventional system based on the introduction of exogenous Cas9 and gRNAs when creating mice with multiple (≤ 10) simultaneous mutations (Fig. 4). We found that the gene-editing ability of maCas9 in sCAT-derived zygotes matched with that observed in WT zygotes electroporated with 100 ng/µL *Cas9* mRNA or 50 ng/µL Cas9 protein and gRNA (Figs. 2c and 3e). We also compared the number of target loci simultaneously genome-edited in the F0 pups generated by these two approaches (introducing 50 ng/µL exogenous Cas9 protein with different numbers of gRNAs into WT zygotes for the conventional system). When maCas9-containing eggs were electroporated, we obtained pups with multiple genome-edited loci (Fig. 4c) and a higher number of mice carrying mutations in 8–9 or 6–7 loci simultaneously per individual (Fig. 4d). Thus, the maCas9-based genome editing system appears to be superior for efficiently generating pups with multiple mutated loci by one-step EP in the presence of gRNAs alone. To assess possible off-target effect induction, we examined the off-target activities in five genes proven to exhibit high on-target activities, finding no appreciable off-target mutations in any of the loci screened (Table S3).

Lastly, we found that the number of delivered pups exhibiting both genome editing at a specific locus and simultaneous genome editing at multiple target loci was higher for the maCas9-based genome editing system than for the conventional method based on the introduction of Cas9 mRNA (or protein) and gRNAs (see Figs. 2c, 3e, 4b, S5c and S7e). The lower birth rate in the latter group may be partly due to toxic components included in the synthesized *Cas9* mRNA and purchased Cas9 protein products, or due to the upper limit of the amount of *Cas9* mRNA/protein introduced into zygotes, because the non-maCas9 zygotes co-transfected with low amounts of Cas9 (15 ng/µL *Cas9* mRNA or 5 ng/µL Cas9 protein) yielded similar numbers of delivered pups to the maCas9 zygotes (Fig. 2c). This property is particularly beneficial because it enables analysis of genome-edited F0 mice during the early stages of experiments (Fig. S8).

There are two potential limitations of the maCas9 method based on the use of sCAT mice. First, because the genetic background of sCAT mice is the (BDF 1 × C57Bl/6J) × C57Bl/6J backcross^30^, it is not possible to create gene-modified mice with another background. Therefore, it will be necessary to create a sCAT strain with another common genetic background, such as 129, or obtain a congenic strain by repeated backcrosses. Second, simultaneous multiple gene modifications in maCas9 zygotes derived from sCAT mice are limited to nine sites (Fig. 4), probably due to the limited amount of maCas9 that can be contained in maCas9 zygotes. Therefore, the creation of a sCAT line with higher Cas9 expression is desirable. Our most important finding, however, is that this novel gene-editing system based on the use of maCas9 considerably facilitates the generation of mice with single and multiple gene modifications and could be applied to various animal species other than mice^16–20^. In addition, similar effects could be obtained using other Cas proteins^41,42^.

In conclusion, maCas9-based genome editing does not require exogenous Cas9 mRNA (or protein) that has to be prepared in-house or purchased from external companies. The method is also beneficial for producing mice carrying mutations in multiple genes and its efficiency in generating indel and KI mutations is comparable to that of the conventional CRISPR/Cas9-based system for generating genome-edited mice, in which exogenous Cas9 mRNA (or protein) and gRNAs are introduced into WT eggs. Moreover, this novel system features lower mosaicism and higher rates of genome-edited pup delivery than the conventional CRISPR/Cas9-based system. Our sCAT mice will be distributed to the RIKEN BioResource Center (RIKEN, BRC, Tsukuba, Japan), from where these could be purchased by researchers around the world for various experimental purposes.

## Methods

### Ethics approval for animal experimentation

All animal care and handling procedures were performed in agreement with the guidelines of the Shinshu University Committee on Recombinant DNA Security and approved by the Animal Care and Experimentation Committee of Shinshu University (permit no. 300044).

### Mice and zygotes

In this study, we used sCAT (systemically Cas9-expressing Tg)^30^ transgenic mouse line and ICR mice. sCAT mice were maintained in-house and PCR-based genotyping for the presence of transgenes was carried out using the Cas9 Tg-1S/Cas9 Tg-1A primer set^30^. ICR female mice (aged 8–10 weeks) were purchased from CLEA Japan (Tokyo, Japan). Fertilized eggs were obtained via a standard IVF protocol described by Sakurai *et al*.^9^ and used for the experiments 6–8 h after insemination (time of insemination = 0 h).

### Preparation of genome editing components

To prepare *EGFPA95* mRNA, fragments containing *EGFP* cDNA, a 95 poly(A) stretch, and *Sap* I sites cloned into the pESA85 vector^27^ were amplified by PCR, and the resultant fragments were cloned into the *Eco* RI sites of pBluescript II (Agilent Technologies Japan, Ltd., Tokyo, Japan) to create pEGFPA95. *EGFPA95* mRNA was then synthesized using the mMessage mMachine T3 kit (Ambion, Life Technologies Japan, Ltd., Tokyo, Japan) with *Sap* I-digested pEGFPA95 as a template. Cas9A95 mRNA was synthesized from *Sap* I-digested pBS-NFCas9^9^ with a 95 poly(A) stretch as a template. Cas9 protein was purchased from Integrated DNA Technologies, Inc. (IDT; Coralville, Iowa, USA). *Et1, Hprt, Klf5*, and *Ramp1* gRNAs (Table S4) were the same as those described by Sakurai *et al*.^30^. *Adm, Amy, ALdh2, Ar*^35^, *Cyp1a1, Npr3*, and *Tyr* gRNAs, and *R3*-gRNA were prepared using the methods described by Sakurai *et al*.^30^. Briefly, the candidate target region for each locus was determined using the CRISPRDirect web server^43^ (Table S4), and the annealed oligonucleotides for each gene were inserted into the gRNA region of pgRNA_GFP-T1 (#41819; Addgene). Using the resultant vector DNA as a template, the 83-bp PCR products spanning the gRNA scaffold and TTTTTT site were subcloned into pBluescript II to obtain a pgRNA vector. Using this vector as a template, PCR products containing the T7 promoter sequence were prepared and used for gRNA synthesis using a MEGAshortscript T7 transcription kit (Ambion). *Klf5* + flagx3 ssODNs (169 bp) and *Ar* + *loxP* ssODNs (138 bp) were obtained from IDT as dried materials. The ssODN sequences are shown in Table S5.

### Electroporation, embryo transfer, and microinjection

Electroporation was performed with a CUY21EDIT electroporator II (BEX Co., Tokyo, Japan) and platinum electrodes set in a plastic plate (#LF501PT1–10; 1 mm gap, 10 mm length, 3 mm width, and 0.5 mm height; BEX Co.) by using the method of Hashimoto and Takemoto^31^ with some modifications. A 5 µL drop containing several sets of genome editing components (gRNAs alone; gRNAs and ssODN; gRNAs and *Cas9* mRNA; gRNAs, *Cas9* mRNA and ssODN; *Cas9* RNPs; and *Cas9* RNPs and ssODN) in a 1:1 mixture of Opti-MEM (Thermo Fisher Scientific K.K., Tokyo, Japan) and 75% phosphate buffered saline was placed between the electrodes and kept under observation with a dissecting microscope. *Cas9* RNPs were prepared by incubating a 1:1 mixture of gRNA(s) and Cas9 protein solutions for 20–30 min at room temperature prior to electroporation. Zygotes (8–30) were placed into the drop, after which EP was carried out at 20 V, switching between on and off for 3 ms and 97 ms, respectively, five times.

Some electroporated eggs were subjected to *in vitro* cultivation in KSOM medium up to the 2-cell or blastocyst stage at 37 °C in conditions of 95% humidity and 5% CO_2_. The remaining embryos were transferred into the oviducts of pseudopregnant ICR females and allowed to develop to the mid-gestational stages (12.5–14.5 dpc) or full-term. Zygote microinjection was performed as described previously^9^ using a Narishige-Olympus microinjection system (MMO-202N, MM-89, UT-2, IM-9B, and IX-70; Narishige Group, Ltd., Tokyo, Japan). *EGFPA95* mRNA (0–200 ng/µL in 75% phosphate buffered saline) was introduced into both the cytoplasm and pronuclei of the zygotes. The injected eggs were cultivated in KSOM *in vitro* up to the 2-cell stage.

### Fluorescence detection and analysis

*EGFPA95* mRNA-derived fluorescence in the electroporated or microinjected embryos was observed using an inverted fluorescence microscope (IX-70; Olympus, Tokyo, Japan) with a U-MWIBA2 filter set (Olympus) and recorded using a DP73 color fluorescence camera (Olympus) under the same exposure and duration conditions. The data were analyzed using ImageJ software (https://imagej.net/User_Guides). Fluorescence was expressed as the average fluorescence in 8–10 embryos per group after deducting background fluorescence intensity.

### Analysis of indel, KI, and mosaicism

In this study, we attempted to perform genome editing on a total of 12 loci (Table S4). PCR primers for amplifying regions spanning the mutated sequences are shown in Table S5. The off-target candidates for *Ar* (Fig. S7), *Cyp1A1* (Fig. 4), *Et1* (Figs. 2 and 4), *Klf5* (Fig. 4)*, Npr3* (Fig. 4), and *Hprt* (Fig. 4) were chosen using CRISPRDirect and the top three candidates were analyzed (Table S6). For blastocyst PCR, crude DNA solution prepared from a single blastocyst^9^ was genotyped for Cas9 transgenes and PCR was performed on the region recognized by the *Et1* gRNA. For obtaining PCR products from mid-gestational fetus or newborn, genomic DNA was isolated from a part of the whole fetus or from a newborn’s ear, respectively. To analyze indels, the PCR products were subjected to the T7 endonuclease I-based assay^9^, direct sequencing, or the Cas9 RNP cut assay (Guide-it Genotype Confirmation kit, Takara Bio Inc., Shiga, Japan).

Successful KI of the FLAG × 3 sequence into the *Klf5* locus and of the *loxP* KI sequence into the *Ar* locus was determined by *Cla* I and *Bam* HI digestion of the PCR products generated by amplifying the region recognized by *Klf5*-gRNA and *Ar*-gRNA, respectively. Three samples in which the FLAG × 3 sequence or *loxP* KI sequence had been knocked-in were randomly selected and used to confirm the correct KI of these sequences into the target locus.

To compare the mosaicism of the *Et1* and *Tyr* alleles, the PCR amplicons of KO samples were cloned into the TA cloning vector pMD20 (Takara Bio Inc., Shiga, Japan) for sequencing, according to the manufacturer’s instructions. Approximately 5–8 plasmids per sample were purified, and the possibly mutated locus was sequenced using a BigDye terminator Cycle Sequencing Kit ver3.1 and an ABI Genetic Analyzer 3130 (Applied Biosystems, Life Technologies Japan, Ltd., Tokyo, Japan). Data were analyzed by using Genetyx-Mac ver.13.0.3 (Software Development Co. Ltd., Tokyo, Japan) and Clustalw (http://www.genome.jp/tools-bin/clustalw). Furthermore, for analysis of the mosaicism in the *Tyr* alleles, the PCR amplicons of KO samples were directly sequenced, and the data obtained were analyzed using ICE2 (Synthego Corporation, Silicon Valley, CA, USA). Samples with ≥3 indels per genome were considered as representatives of mosaic individuals in this study.

### Statistical analysis

Statistical differences between the experimental and control groups were calculated using two-sided Fisher’s exact tests for a 2 × 2 and 2 × 3 Contingency Table. Differences were considered to be statistically significant if *P* < 0.05.

## Supplementary information

Dataset1.

## Data Availability

sCAT mice are currently being deposited with the RIKEN BioResource Center (RIKEN, BRC, Tsukuba, Japan; URL: https://ja.brc.riken.jp/), and will be available from the RIKEN BRC in the near future.
