# Supplementary material for: Production of genetically engineered mice with higher efficiency, lower mosaicism, and multiplexing capability using maternally expressed Cas9
Source: Sci Rep. 2020 Jan 23;10:1091. doi: 10.1038/s41598-020-57996-7 (PMC6978307; doi:10.1038/s41598-020-57996-7)

Supplementary Dataset for

Scientific Reports

Production of genetically engineered mice with higher efficiency, lower mosaicism, and multiplexing capability using maternally expressed Cas9

Takayuki Sakurai[1, 2, *], Akiko Kamiyoshi[1, 2], Hisaka Kawate[1, 2], Satoshi Watanabe[3], Masahiro Sato[4] and Takayuki Shindo[1, 2]

1. Department of Life Innovation, Institue for Biomedical Sciences, Shinshu University, 3-1-1 Asahi, Matsumoto, Nagano 390-8621, Japan

2. Department of Cardiovascular Research, School of Medicine, Shinshu University, 3-1-1 Asahi, Matsumoto, Nagano 390-8621, Japan

3. Animal Genome Research Unit, Division of Animal Science, National Institute of Agrobiological Sciences, Ibaraki 305-8602, Japan

4. Section of Gene Expression Regulation, Frontier Science Research Center, Kagoshima University, 8-35-1 Sakuragaoka, Kagoshima, Kagoshima 890-8544, Japan

* Correspondence: e-mail: tsakurai@shinshu-u.ac.jp

Supplementary Figs. S1 to S9

Supplementary Tables S1 to S6

Supplementary Information (the full-length gel data)

Supplementary Figures

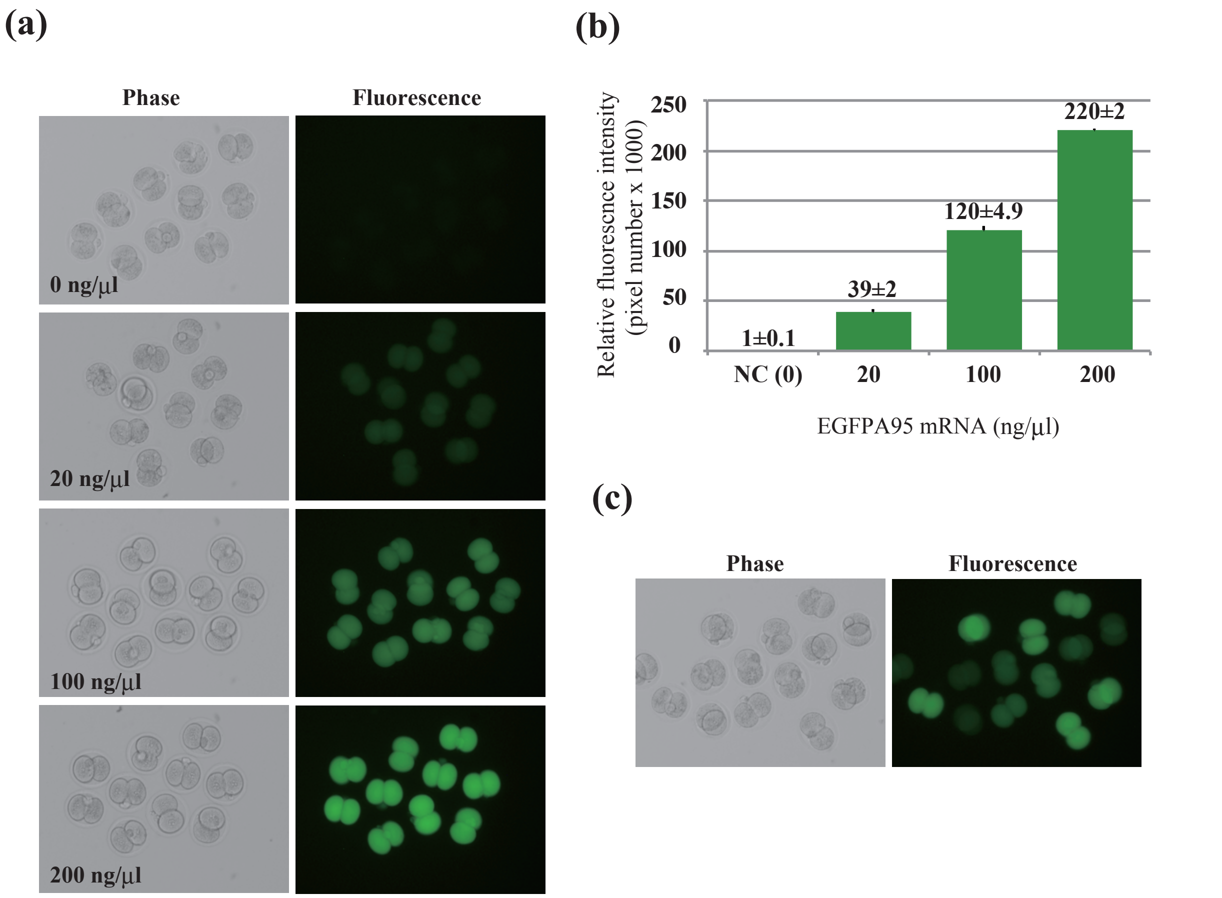

Figure S1. In vitro electroporation of zygotes in the presence of synthetic EGFPA95 mRNA. (a) Phase and fluorescence-field images of 2-cell embryos developed from zygotes electroporated with various amounts of EGFPA95 mRNA. (b) Relative fluorescence intensity of 2-cell embryos developed from the electroporated zygotes. Data are presented as the mean ± S.E.M. (c) Phase and fluorescence-field images of 2-cell embryos developed from zygotes obtained after the microinjection of 20 ng/µL EGFPA95 mRNA.

(a)

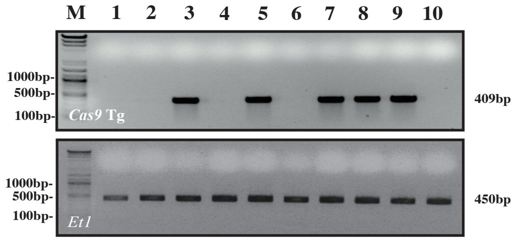

(b)

	Sequences around *Et1* target region	Indel	Frequency
Wt:	ccataatagGCGCGTCGTACCGTATGGACTGGgaggttcttccaggtccaagcgtt		
1:	ccataatagGCGCGTCGTACCGTATGGACTGGgaggttcttccaggtccaagcgtt	Intact sequence	6/6
2:	ccataatagGCGCGTCGTACCGTATG-ACTGGgaggttcttccaggtccaagcgtt	1 bp deletion	2/5
	ccataatagGCGCGTCGTACCGTATGGACTGGgaggttcttccaggtccaagcgtt	Intact sequence	3/5
3:	ccataatagGCGCGTCGTACCGTATG--CTGGgaggttcttccaggtccaagcgtt	2 bp deletion	1/5
	ccataatagGCGCGTCGTACCGTATGGACTGGgaggttcttccaggtccaagcgtt	Intact sequence	4/5
4:	ccataatagGCGCGTCGTACCGTAT----TGGgaggttcttccaggtccaagcgtt	4 bp deletion	1/5
	ccataatagGCGCGTCGTACCGTATGGACTGGgaggttcttccaggtccaagcgtt	Intact sequence	4/5
5:	ccataatagGCGCGTCGTACCGTATG-----Ggaggttcttccaggtccaagcgtt	5 bp deletion	1/6
	ccataatagGCGCGTCGTACCGTATGGACTGGgaggttcttccaggtccaagcgtt	Intact sequence	5/6
6:	ccataatagGCGCGTCG---------GACTGGgaggttcttccaggtccaagcgtt	9 bp deletion	1/6
	ccataatagGCGCGTCGTACCGTATGGACTGGgaggttcttccaggtccaagcgtt	Intact sequence	5/6
7:	ccataatagGCGCGTCGTACC----------Ggaggttcttccaggtccaagcgtt	10 bp deletion	2/4
	ccataatagGCGCGTCGTACCGTATGGACTGGgaggttcttccaggtccaagcgtt	Intact sequence	2/4
8:	ccataatagGCGCGTCGTACCGTAT-----------ttcttccaggtccaagcgtt	11 bp deletion	3/6
	ccataatagGCGCGTCGTACCGTATGGACTGGgaggttcttccaggtccaagcgtt	Intact sequence	3/6
9:	ccataatagGCGCGTCGTACC-------CTGGgaggttcttccaggtccaagcgtt	7 bp deletion	3/6
	ccataatagGCGCGTCGTAC--------CTGGgaggttcttccaggtccaagcgtt	8 bp deletion	3/6
10:	ccataatagGCGCGTCGTACCGTATG--CTGGgaggttcttccaggtccaagcgtt	2 bp deletion	1/6
	ccataatagGCGCGTCGTACCGTA----CTGGgaggttcttccaggtccaagcgtt	4 bp deletion	2/6
	ccatatgacgggc-------------------aggttcttccaggtccaagcgtt	20 bp indel	1/6
	ccataatagGCGCGTCGTACCGTATGGACTGGgaggttcttccaggtccaagcgtt	Intact sequence	1/6

Figure S2. Characterization of the blastocysts carrying indels in the *Et1* locus that developed from sCAT zygotes after electroporation in the presence of 25 ng/μL *Et1*-gRNA shown in Fig. 1.
(a) Upper panel, agarose gel electrophoresis results of genotyping for the Cas9 gene. Lower panel, agarose gel electrophoresis of PCR products corresponding to the sequences around the sites targeted by *Et1*-gRNA. Lanes 1–10, PCR products amplified from genomic DNA of each blastocyst; M, lambda *Hin*dIII + 100-bp ladder markers. The images of these full-length gels are presented in Supplementary Information Figure S2(a)
(b) Sequencing results of *Et1* PCR products shown in lanes 1–10 in (a). Wt, wild-type sequence around the sites targeted by *Et1*-gRNA. Blue color indicates nucleotides corresponding to the gRNA target sequence. Red color indicates the protospacer adjacent motif site. Dashed lines indicate the deletion of nucleotide(s). Frequency shows the ratio (in %) of the number of clones detected to the total number of clones sequenced.

(a)

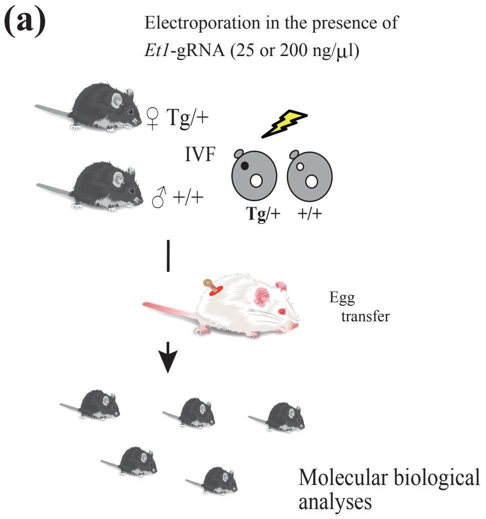

(b)

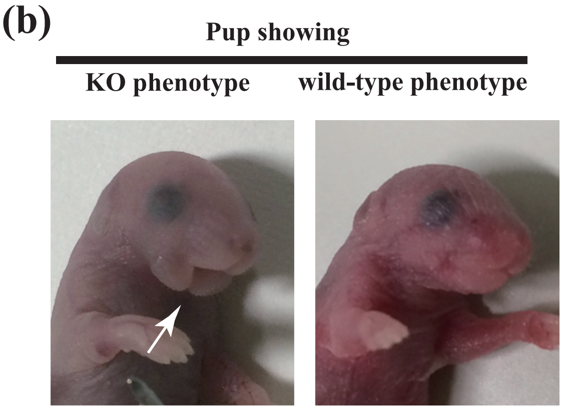

(c)

CRISPR reagents	No. zygotes electroporated	No. zygotes transferred	No. pups obtained: Total (%)	No. pups obtained: Genotype for Cas9 transgene	No. pups with: KO phenotype	No. pups with: wild-type phenotype	Frequency (%): KO	Frequency (%): Indels
Et1-gRNA (25 ng/μl)	50	50	22 (44)	+/+	0	15	0/15 (0)	10/15 (67)
				Tg/+	0	7	0/7 (0)	4/7 (57)
Et1-gRNA (200 ng/μl)	40	40	23 (58)	+/+	4	10	4/14 (29)	14/14 (100)
				Tg/+	5	4	5/9 (56)	9/9 (100)

Figure S3. Correlation between *Et1*-gRNA concentration used and the rate of *Et1* KO phenotype occurrence.
(a) Schematic of the experimental procedure. (b) Representative newborn pups exhibiting *Et1* KO and wild-type phenotypes. White arrow in the left panel (KO phenotype) indicates craniofacial abnormality.
(c) Table summarizing the occurrence rate of the pups with KO phenotype that developed from sCAT zygotes after electroporation in the presence of 25 ng/μL or 200 ng/μL *Et1*-gRNA.

	Sequences around *Et1* target region	Indel	Frequency
Wt:	ccataatagGCGCGTCGTACCGTATGGACTGGgaggttcttccaggtccaagcgtt		
1:	ccataatagGCGCGTCGTACCGTA--GACTGGgaggttcttccaggtccaagcgtt	2 bp deletion	5/5
2:	ccataatagGCGCGTCGTACCGTATGgtGACTGGgaggttcttccaggtccaagcgtt	2 bp insertion	8/8
3:	ccataatagGCGCGTCGTACCGTATG-ACTGGgaggttcttccaggtccaagcgtt	1 bp deletion	2/6
	ccataatagGCGCGTCGTACCG-------TGGgaggttcttccaggtccaagcgtt	7 bp deletion	4/6
4:	ccataatagGCGCGTCGTACCGTATGaggTGGgaggttcttccaggtccaagcgtt	3 bp indel	2/8
	ccataatagGCGCGTtcttcca-------TGGgaggttcttccaggtccaagcgtt	14 bp indel	6/8
5:	ccataatagGCGCGTCGTACCG-----ACTGGgaggttcttccaggtccaagcgtt	4 bp deletion	4/6
	ccataatagGCGCGTCGTACCGTA-GGACTGGgaggttcttccaggtccaagcgtt	1 bp deletion	2/6
6:	ccataatagGCGCGTCGTACCGTATGGgACTGGgaggttcttccaggtccaagcgtt	1 bp insertion	3/7
	ccataatag------------------------------------gtccaagcgtt	36 bp deletion	4/7
7:	ccataatagGCGCGTCGTACCattgg---TGGgaggttcttccaggtccaagcgtt	8 bp indel	1/8
	ccataatagGCGCGTCGTACCa----GACTGGgaggttcttccaggtccaagcgtt	5 bp indel	7/8
8:	ccataatagGCGCGTCG-------------------ttcttccaggtccaagcgtt	19 bp deletion	3/6
	ccataatagGCGCGT---------------------ttcttccaggtccaagcgtt	21 bp deletion	3/6
9:	ccataatagGCGCGTCGTACCGTATGtGACTGGgaggttcttccaggtccaagcgtt	1 bp insertion	2/8
	ccataatagGCGCGTCGTACCG--TGGACTGGgaggttcttccaggtccaagcgtt	2 bp deletion	3/8
	ccataatagGCGCGTC-------------TGGgaggttcttccaggtccaagcgtt	13 bp deletion	3/8
10:	ccataatagGCGCGTCGTACC----------Ggaggttcttccaggtccaagcgtt	10 bp deletion	3/6
	ccataatagGCGCGTCGTACC--------------gttcttccaggtccaagcgtt	14 bp deletion	1/6
	ccataatagGCGCGTCGTACCGTATG-ACTGGgaggttcttccaggtccaagcgtt	1 bp deletion	2/6

Figure S4. Nucleotide sequences around the sites targeted by *Et1*-gRNA in pups nos. 1–10 with wild-type Cas9 genotype and *Et1* KO phenotype that developed from sCAT zygotes after electroporation in the presence of 200 ng/μL *Et1*-gRNA as shown in Fig. 2c. Wt shows wild-type sequence around the sites targeted by *Et1*-gRNA. Blue color indicates nucleotides corresponding to gRNA target sequence. Red color indicates the protospacer adjacent motif site. Dashed lines indicate the deletion of nucleotide(s). Frequency shows the ratio (in %) of the number of clones detected to the total number of clones sequenced.

(a)

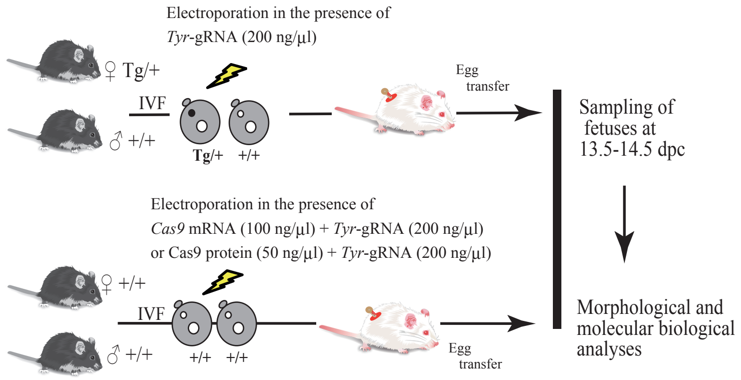

(b)

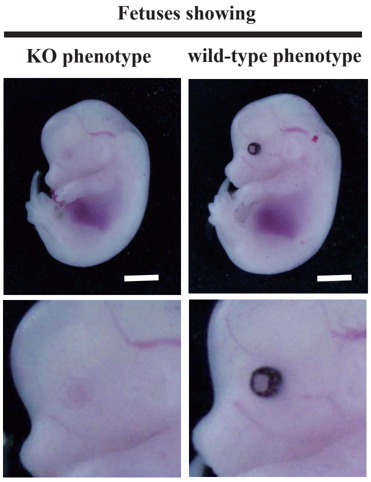

(c)

IVF pairs ♀ ♂	CRISPR reagents	No. zygotes electroporated	No. zygotes transferred	No. fetuses at 13.5-14.5 dpc obtained: Total (%)	No. fetuses with KO phenotype	No. fetuses with wild-type phenotype	Frequency (%) KO	Frequency (%) Indels
Tg/+ +/+	*Tyr*-gRNA (200 ng/µl)	55	55	32 (58)	15	17	15/32 (47)	24/32(75)
Control +/+ +/+	*Cas9* mRNA (100 ng/µl) + *Tyr*-gRNA (200 ng/µl)	56	56	25 (45)	12	13	12/25 (48)	20/25(80)
Control +/+ +/+	Cas9 protein (50 ng/µl) + *Tyr*-gRNA (200 ng/µl)	60	60	19 (32)	13	6	13/19 (68)	16/19(84)

(d) No. indels per genome

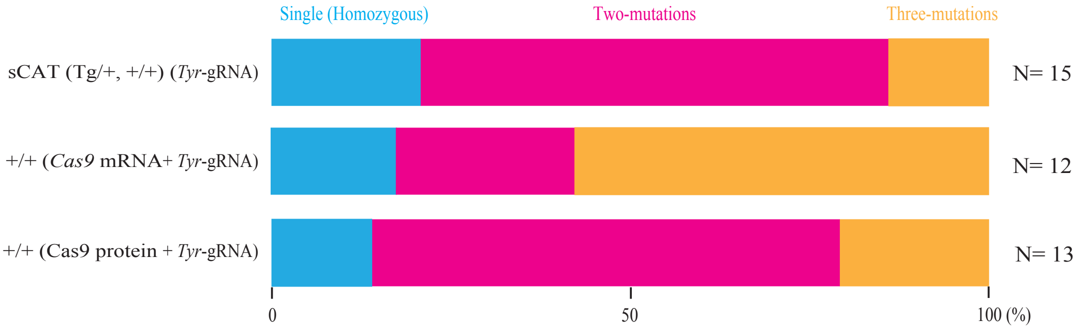

Figure S5. Comparison of *Tyr* gene editing efficiency in maCas9 and wild-type zygotes.
(a) Schematic of the experimental procedure for examining the efficiency of *Tyr* gene editing in maCas9 and wild-type (non-maCas9) zygotes. (b) Representative 14.5 dpc fetuses exhibiting *Tyr* KO and wild-type phenotypes. Left panel (KO phenotype): eye pigment deficiency. Right panel (WT phenotype): normal eye pigment. Scale bars indicate 1.8 mm. (c) Table summarizing the efficiency of *Tyr* gene editing observed in maCas9 and wild-type zygotes after transfection with exogenous Cas9 mRNA (or protein) and gRNA.
(d) Number of indels per genome of *Tyr* KO fetuses developed from maCas9 zygotes electroporated with 200 ng/μL *Tyr* -gRNAs only and non-maCas9 zygotes electroporated with 200 ng/μL *Tyr* -gRNAs + 100ng/μL Cas9 mRNA or 200 ng/μL *Tyr* -gRNAs + 50ng/μL Cas9 protein.

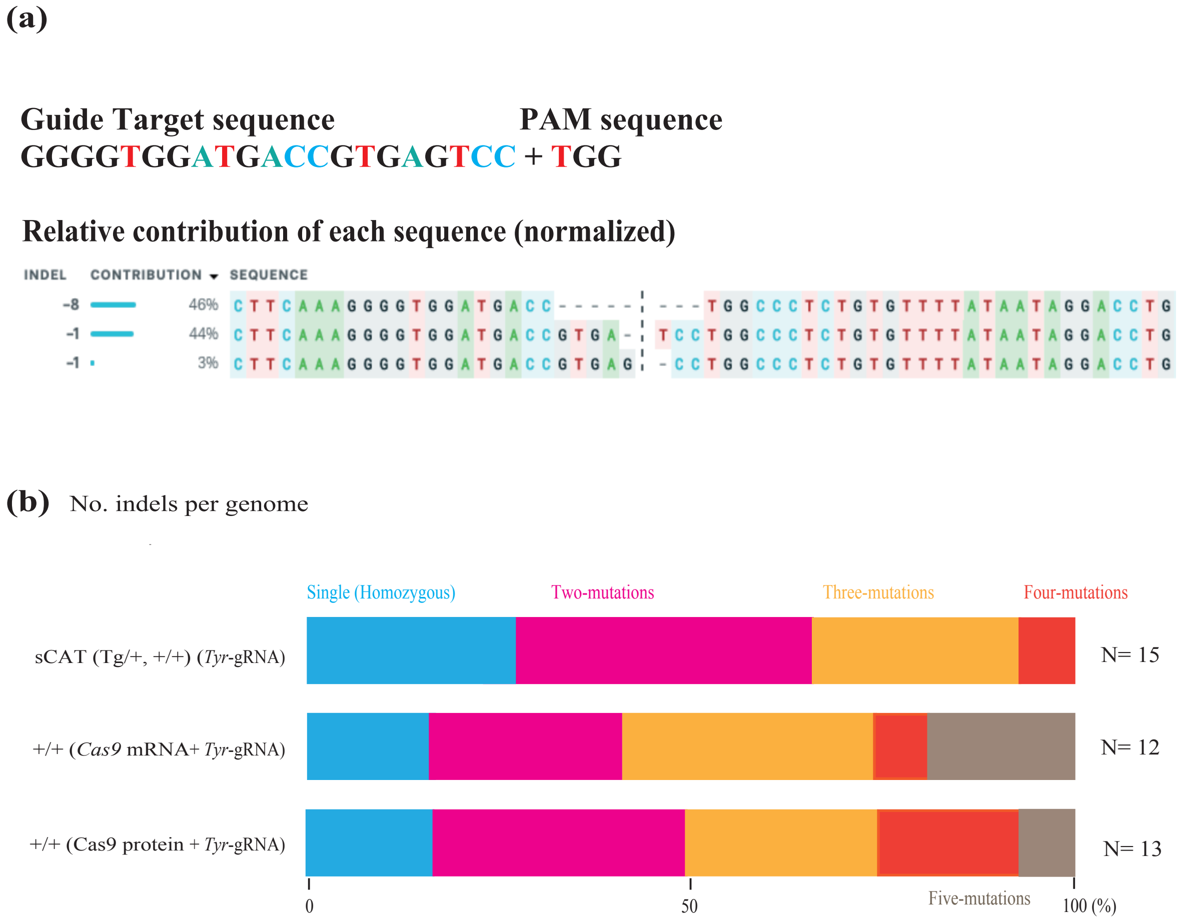

Figure S6. Indel analysis of *Tyr* KO fetuses developed from maCas9 zygotes and non-maCas9 zygotes by ICE2. (a) A representative result. Upper part shows the guide target sequence and protospacer adjacent motif sequence of *Tyr* gene, and the lower part shows relative indel contribution of a *Tyr* KO fetus developed from maCas9 zygotes. (b) Number of indels per genome in *Tyr* KO fetuses developed from maCas9 zygotes and non-maCas9 zygotes. The samples used are the same as those used for TA cloning analyses (see Fig. S5d). Samples with indel frequency > 5% were counted.

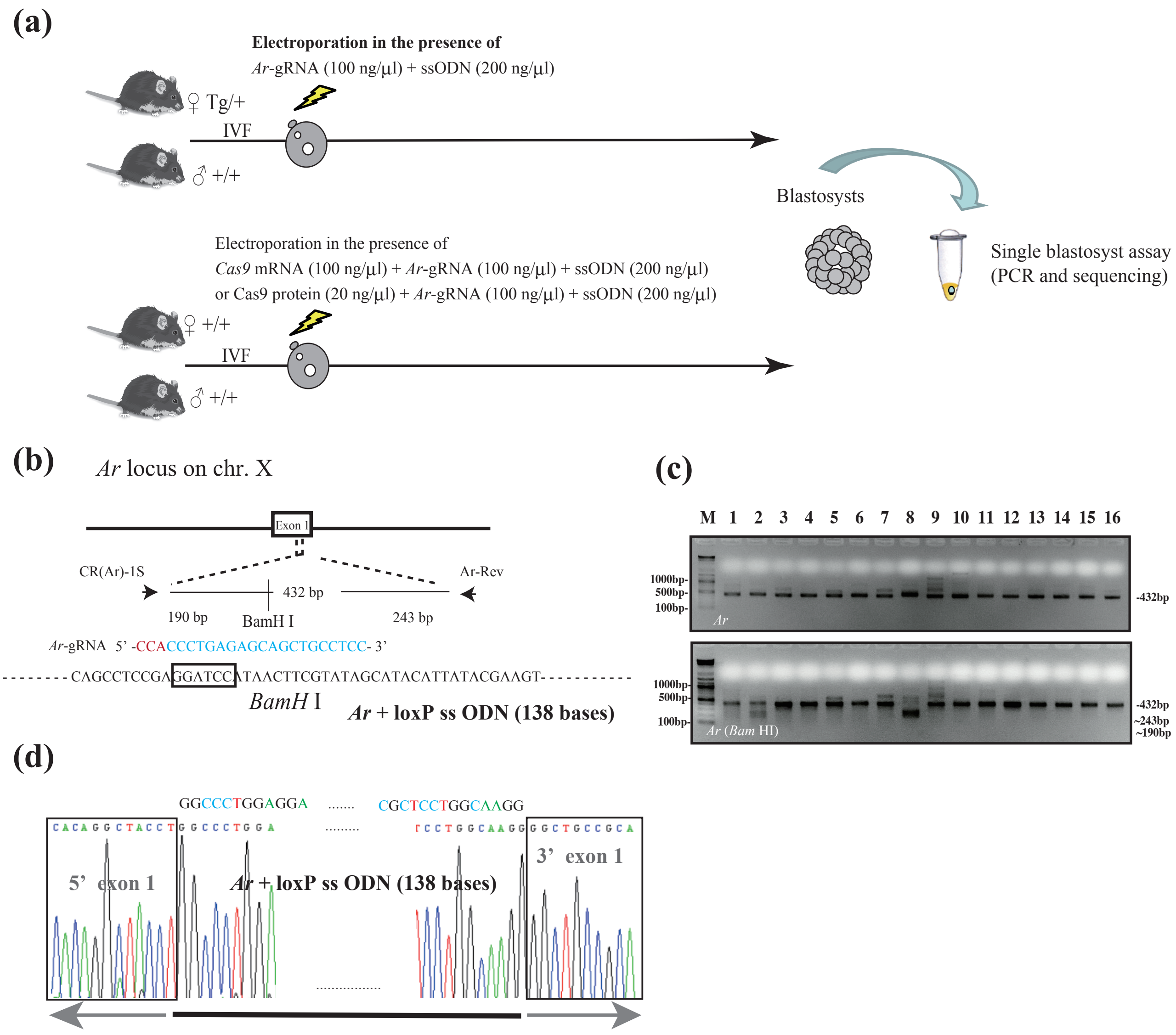

IVF pairs ♀ ♂	CRISPR reagents	No. zygotes electroporated	No. blastocysts developmed (%)	Frequency (%) KI	Frequency (%) Indels
Tg/+ +/+	*Ar*-gRNA ssODN	55	38 (69)	3/38 (8)	29/38 (76)
Control +/+ +/+	*Ar*-gRNA ssODN *Cas9* mRNA	54	8 (15)	0/8 (0)	6/8 (75)
Control +/+ +/+	*Ar*-gRNA ssODN Cas9 protein	49	4 (8)	0/4 (0)	3/4 (75)

Figure S7. Comparison of knock-in (KI) mutation efficiency in maCas9 and wild-type zygotes.
(a) Schematic of the experimental procedure for examining KI mutation efficiency using *Ar* + loxP ssODN targeting the *Ar* gene. (b) *Ar*-gRNA targeting exon 1 of the murine *Ar* gene. The sequence recognized by *Ar*-gRNA is shown in blue and the protospacer adjacent motif sequence is shown in red. Arrows indicate the locations of the PCR primers (see Table S5). All *Ar* + loxP ssODN ssODN sequences are shown in Table S5. (c) Representative image of agarose gel electrophoresis of PCR products amplified from the genomic DNA of pups (1–16) obtained from maCas9 zygotes electroporated with 100 ng/μL *Ar*-gRNAs and 200 ng/μL ssODN. Upper and lower panels show the results of the restriction fragment length polymorphism assay using the *Bam* HI enzyme. Pups 2 and 8 harbored the KI mutation. Lanes 1–16 show PCR products amplified from the genomic DNA of all pups; M, lambda *Hin*dIII + 100-bp ladder markers. Full-length gel images are presented in Supplementary Information Figure S7. (d) Sequences of 5′ and 3′ junction sites in pup 8. Sequence chromatograms show the correct KI of *Ar* + loxP ssODN (Table S5) into exon 1 of the *Ar* gene.
(e) Table comparing KI mutation induction efficiency by maCas9-based genome editing and genome editing in wild-type zygotes after transfection in the presence of exogenous Cas9 mRNA (or protein) and gRNA.

(a) **Electroporation in the presence of *Et1*-gRNA (20 ng/µl), *R1*-gRNA (100 ng/µl) and *R3*-gRNA (100 ng/µl)**

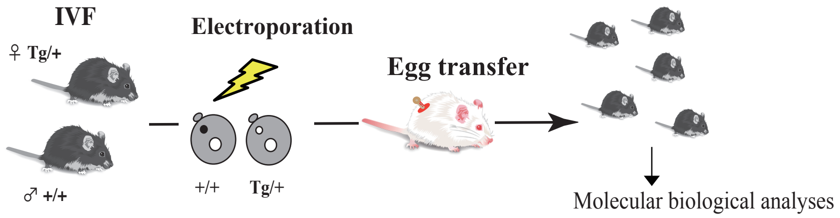

(b)

Electroporation					No. fetuses with Indels (%)		
No. zygotes	No. transferred	No. fetuses obtained (%)	No. fetuses with *Cas9* genotype		*Et 1*	*Ramp 1*	*Ramp 3*
86	86	37(43)	37		21 (57)	27 (73)	22 (59)
			16	Tg/+	8 (50)	10 (63)	7 (44)
			21	+/+	13 (62)	17 (81)	15 (71)

(c)

No. ♂ picked up randomly	Genotype of *Cas9* transgene	*Et1* phenotype	Indels analyzed *		
			Et1	*Ramp 1*	*Ramp 3*
1	W	Normal	YES	YES Homo	YES Homo
2	W	Normal	W	W	YES
3	Tg/+	Normal	W	W	W
4	Tg/+	Normal	YES	YES	YES
5	Tg/+	Normal	YES	YES	W
6	Tg/+	Normal	W	YES	YES
7	W	Normal	W	YES Homo	YES
8	W	Normal	W	YES	W
9	Tg/+	Normal	W	YES	YES Homo
10	Tg/+	Normal	W	YES Homo	YES
11	W	Normal	W	YES Homo	YES
12	Tg/+	Normal	W	YES	YES
13	Tg/+	Normal	YES	YES Homo	YES
14	W	Normal	W	YES	YES
15	Tg/+	Normal	W	YES	W

Figure S8. Generation of gene-modified mice with simultaneous disruption of three genes (*Et1*, *Ramp1*, and *Ramp3*). (a) Schematic of the experimental procedure. (b) Table summarizing the numbers of F0 pups developed from sCAT zygotes after electroporation in the presence of the three gRNAs targeted to *Et1*, *Ramp1*, and *Ramp3*. (c) Table summarizing Cas9 genotypes and indel presence in the three genes in 15 male pups randomly selected from a group of 37 pups mentioned in (b).

(a)

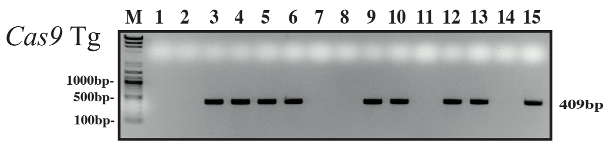

(b)

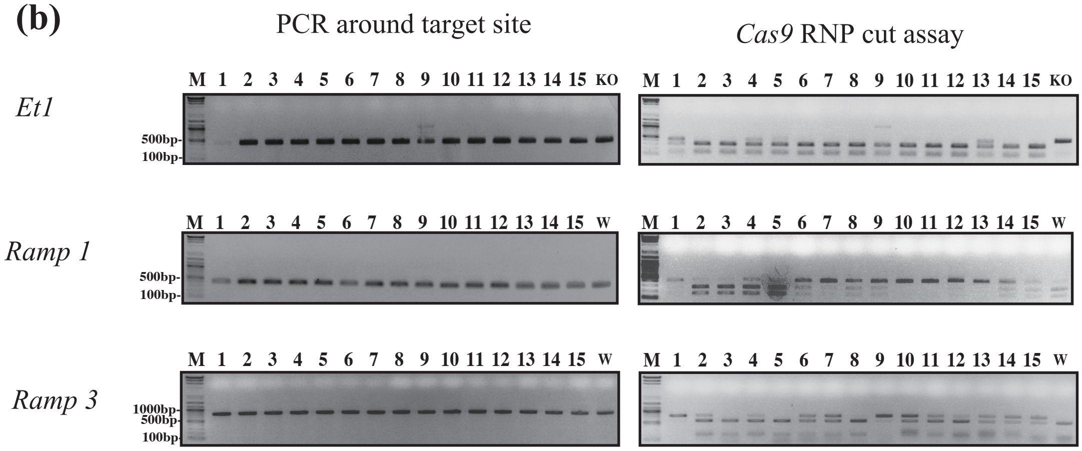

Figure S9. Images of gels from the experiments described in Figure S4c.
(a) *Cas9* genotyping by agarose gel electrophoresis. (b) Detection of indels in *Et1, Ramp1,* and *Ramp3* genes by agarose gel electrophoresis. Left three panels show PCR products amplified from genomic DNA of randomly picked male pups. Top right panel shows the result of the Cas9 ribonucleoprotein (RNP) cut assay using PCR products corresponding to the sequences around *Et1* target sites. Band pattern in lanes 1, 4, 5 and 13 indicates pups with heterozygous indels, whereas band pattern in other lanes indicates wild-type pups. Lane KO indicates the pup homozygous for the indels (used as positive control).
Middle right panel shows the result of the Cas9 RNP cut assay using PCR products corresponding to the sequences around *Ramp1* target site. Lanes 1, 7, 10, 11 and 13 indicate pups with homozygous indels.
Lanes 4, 5, 6, 8, 9, 12, 14, and 15 indicate pups with heterozygous indels. Lanes 2 and 3 indicate wild-type pups.
Line W indicates a wild-type pup (used as positive control).
Bottom panel shows the result of the Cas9 RNP cut assay using PCR products corresponding to the sequences around *Ramp3* target site. Lanes 1 and 9 indicate pups with homozygous indels. Lanes 2, 4, 6, 7, 10, 11, 12, 13, and 14 indicate pups with heterozygous indels. Lanes 3, 5, 8 and 15 indicate wild-type pups.
Line W indicates a wild-type pup (used as positive control). Lanes 1–15, PCR products amplified from genomic DNA of randomly selected male pups nos. 1–15 (shown in Fig. S6c); M, lambda *Hin*dIII + 100-bp ladder markers. The images of these full-length gels are presented in Supplementary Information Figures S9 (a) and (b).

Supplementary Tables

Table S1. Summary of the results of *Et1* gene off target analysis.

Genomic potition (PCR primers)	sequences*	Mutation frequency in 12.5-13 dpc KO fetus (no. mutated embryos/no. total embryos tested) **	
		+/+	Tg/+
Chr2:53269587-53269609 (Et1 Offt1-1/2)	ATAGAGGTTACCGTATGAACTGG	0/12	0/9
Chr6:46674445-46674467 (Et1 Offt2-1/2)	AATCCTTAGACCGTATGGACTGG	0/12	0/9
Chr14:67277916-67277938 (Et1 Offt3-1/2)	TATGAAGTTACCGTTTGGACTGG	0/12	0/9

* Sequences of off-target candidates were determined using the CRISPR designe tool (http://crisprdirect).

* * 12 +/+ fetus and 9 Tg/+ fetus were shown in Fig.3c. These fetus were obtained from the experiment of sCAT zygotes electroporated in the presence of *Et1*-gRNAs (200 ng/μl). sCAT zygotes were prepaired by IVF (♀Tg/+ x ♂+/+).

Table S2. Summary of the results of *Ar* gene off target analysis.

Genomic potition (PCR primers)	sequences*	Mutation frequency in blastcysts obtained (no. mutated blastocyst /no. total blastocyst tested) **
Chr11:3140158-3140172 (Ar OST1-1/2)	CCTCCCTGAGGGCAGCTGCCTCC	3/38
Chr8:121017073-121017287 (Ar OST3-1/2)	CCACCCTGAGAGCAGCAGCCTCT	1/38

* Shen N. et al., Nature Methods 11, 399-402 (2014)

* * 35 blastocysts were shown in Fig.S5e. These blastocysts were obtained from the experiment of sCAT zygotes electroporated in the presence of *Ar*-gRNAs (100 ng/μl). sCAT zygotes were prepaired by IVF (♀Tg/+ x ♂+/+).

Table S3. The results of the off-target analysis of simultaneous multiple gene modified mice*

Target gene	Potition of posibleoff-target sites (PCR primers)	sequences*	Mutation frequency in new born pups (no. mutated pups/ no. total pups tested) **
Cyp1A1	Chr6:28284885-28284899 (Cyp1 Offt1-1/2)	TGGTAGCAGTAAGTGTACTTAGG	0/35
	Chr9:57443685-57443699 (Cyp1 Offt2-1/2)	TCATTGGAGTACGTGTAGTTGGG	0/35
	Chr12:53607465-53607479 (Cyp1 Offt3-1/2)	GGTAAGTGGTACGTGTACTTCAG	0/35
Et 1	Chr2:53269587-53269609 (Et1 Offt1-1/2)	ATAGAGGTTACCGTATGAACTGG	0/35
	Chr6:46674445-46674467 (Et1 Offt2-1/2)	AATCCTTAGACCGTATGGACTGG	0/35
	Chr14:67277916-67277938 (Et1 Offt3-1/2)	TATGAAGTTACCGTTTGGACTGG	0/35
Hprt	Chr1:16565645-16565655 (Hprt Offt1-1/2)	CCCTCATGCCGAGCGTGCACTCA	0/35
	Chr3:121103025-121103035 (Hprt Offt2-1/2)	CCATCATGCCGGGGAAAGCATCT	0/35
	Chr9:107452508-107452518 (Hprt Offt3-1/2)	CCCTCATGCCGGGTCTCAGTAAG	0/35
Klf 5	Chr4:46452023-46452037 (Klf5 Offt1-1/2)	CCTTGCCCACGCGGGCTCCCCGG	0/35
	Chr11:102467168-10246718 (Klf5 Offt2-1/2)	CCCTGCCCACGCGGGGCTCTTGG	0/35
	Chr3:96197318-96197328 (Klf5 Offt3-1/2)	CCCTGCCCACGCACCCCAGACAA	0/35
Npr3	Chr7:36674630-36674644 (Npr3 Offt1-1/2)	CCAGTGTGCCAGTACTGGAGAGG	0/35
	Chr9:112044171-112044185 (Npr3 Offt2-1/2)	CCAGTGTGAGAGTACACAATTTG	0/35
	Chr1:43468068-43468082 (Npr3 Offt3-1/2)	CCTGTGTGCGAGCACTACAGATA	0/35

* 35 pups were shown in Fig.5. These pups were obtained from the experiment of sCAT zygotes electroporated in the presence of *10* gRNAs (each 25 ng/μl). sCAT zygotes were prepaired by IVF (♀Tg/+ x ♂+/+).

* *Sequences of off-target candidates were determined using the CRISPR designe tool (http://crisprdirect).

Table S4. gRNA sequences used in this study.

ID	sequence (5' - 3')	Location	GeneBank#
Adm-gRNA	CCGGCGCCGGCGTTCCCTGCTGG	*Adm* exon 4	NM009627
Aldh2-gRNA	GCTGGGCGAGTATGGCCTGCAGG	*Aldh2* exon 12	NM001308450
Ar-gRNA	CCACCCTGAGAGCAGCTGCCTCC	*Ar* exon 1	NM013476
Cyp1a1-gRNA	TGGAGCTGGTACGTGTACTTTGG	*Cyp1a1* exon 3	NM001136059
Et1-gRNA	GCGCGTCGTACCGTATGGACTGG	*Et-1* exon 3	NM010104
Hprt-gRNA	CCGTCATGCCGACCCGCAGTCCC	*Hprt* exon1	NM013556
Iapp-gRNA	CCAACGTGGGATCGAATACATAT	*Iapp* exon3	NM010491
Klf5-gRNA	CCATGCCCACGCGGGTGCTGACC	*Klf5* exon 1	NM009769
Npr3-gRNA	CCCGTGTGCGAGTACGCGGCGGC	*Npr3* exon 1	NM001039181
R1-gRNA	GCTCTGCTTGCCATGGCCCCGGG	*Ramp1*exon 1	NM001168392
R3-gRNA	CCTCTGCAGGTATTATGAAAGCT	*Ramp3* exon 3	NM019511
Tyr-gRNA	GGGTGGATGACCGTGAGTCCTGG	*Tyr* exon 1	NM001317397

The PAM sequece is shown in red.

Table S5. PCR primers and ssODN sequences used in this study.

ID	Sequence (5' - 3')
Cas9 Tg-1S	GTTGTTATAATCTTCTAACAGTAAC
Cas9 Tg-1A	GCGGAACTCCATATATGGGCTATGAACTAATG
CR(Adm)-1S	CTACAAGCCAGGTAACTATGCTCTC
CR(Adm)-1A	GTATCAGCCTGTAAGTCTCGGTTTC
CR(Aldh2)-1S	GGATTATGTAACAAGTTCTAGGACAGC
CR(Aldh2)-1A	TTGTGGGTAAGATCCTAGAGACTGTAT
CR(Ar)-1S	CCTGAGGCCGCTAACATAGCAC
CR(Ar)-Rev	GCTGCTGAAGAAGTTGCATGGTG
CR(Cyp1a1)-1S	TAACTAACAGTGTCAGGCTCTGTGC
CR(Cyp1a1)-2A	ATCTCTGCCAATCACTGTGTCTAC
CR(Et1)-2S	TACAAAGCAGAAGCCCAAACAGAGGTTG
CR(Et1)-2A	GTTCTTTTCCTGCTTGGCAGAAATTCCA
CR(Hprt)-1S	TGAGCCATTGCTGAGGCG
CR(Hprt)-1A	CGCGCCTGATCCTTCCTG
CR(Iapp)-1S	CGGTGAAGGGTGTTGTGTGA
CR(Iapp)-1A	ACACAGTCATCAAGCACAAGC
CR(Klf5)-1S	GGTACGCGCTCTTCTTAGGTT
CR(Klf5)-1A	GCGTGTTTCAGATCGTCTCC
CR(Npr3)-1S	GTCTTTGGTCTCGTCGAAATTGTAG
CR(Npr3)-1A	AAAGATCGAGGTGCTTGTTCTATTG
CR(R1)-1S	CCCACAGGATAGCTGAGGAAC
CR(R1)-1A	CAAGCTGCCCATTGTATGATC
CR(R3)-1S	TGGGGAGGTCTGTTGAAGAAC
CR(R3)-1A	CATAGCCACAGTCAGCACGAC
CR(Tyr)-1S	GTTTTGTATTGCCTTCTGTGGAGT
CR(Tyr)-1A	GGGATGACATAGACTGAGCTGATAG
Ar loxP ssODN	GGCCCTGGAGGAGGAACAGCAGCCTTCACAGCAGCAGG<br>CAGCCTCCGAGGATCCATAACTTCGTATAGCATACATTA<br>TACGAAGTTATTGAGAGCAGCTGCCTCCCCGAGCCTGGG<br>GCGGCCACCGCTCCTGGCAAGG
Klf5 FLAG ssODN	GGATCGCGATCGCTCCGTGTCCCGCTCCCGTAATCCCCAG<br>ACCGTCCATGGACTACAAAGACCATGACGGTGATTATAAA<br>GATCATGACATCGATTACAAGGATGACGATGACAAGGGAC<br>CCACGCGGGTGCTGACCATGAGCGCCCGCCTGGGACCACTG<br>CCCCAGCC

Table S6. PCR primers for off-target analyses used in this study.

ID	Sequence (5’ - 3’)
Ar OST1-S2	GCAGATGGCTGGATATGCTCTTA
Ar OST1-A2	CATCACAAGGAAGACCAAAGAGG
Ar OST3-S2	TATAGATGGACAGACGGGCAGAC
Ar OST3-A2	TCCTGCCACTTCCTCTACTCTGA
Cyp1 Offt1-1	GTACCAAGGCTCACAGCCAGAC
Cyp1 Offt1-2	TACCTTTGTGGGTTCAGCAGGA
Cyp1 Offt2-1	TCTCACAAGGGGACATAGGC
Cyp1 Offt2-2	CATTCCCTGTGGTTGCTTCT
Cyp1 Offt3-1	AGGCCCTTGGTCTTTTGAAG
Cyp1 Offt3-2	GCCAGCCATTCCATTTTACA
Et1 Offt1-1	CACCTATTGCTTGTAGGTCTTCATC
Et1 Offt1-2	GGCTATTATTAGTTGGTTCCCAGAG
Et1 Offt2-1	TAAGATGTGTCTGGTGTCTTCCGTA
Et1 Offt2-2	GAGGAAGAGACAGCTTAGGAGTGAT
Et1 Offt3-1	TTGTCTGATTTCTGCTACTGAACTTG
Et1 Offt3-2	GGGAAAATTGCTCTCTAAACTGTTAC
Hprt Offt1-1	AGTATCGGCACGAGGGACTGA
Hprt Offt1-2	TAGCCTTTCCATCCCAGCCGA
Hprt Offt2-1	ACACAGGTTACTTTGTGCGCT
Hprt Offt2-2	TTCCAAGGATCGGTCTAGAAATGC
Hprt Offt3-1	AAGCTCAGGCCTCCCTCTCTC
Hprt Offt3-2	GCTCCCCCTATGGACGTCAG
Klf5 Offt1-1	GGTGTTCGGGTCCCTGAG
Klf5 Offt1-2	CTCACCCGGCGGAGGAAG
Klf5 Offt2-1	GCCCAGGCTTGCCTTGTATGT
Klf5 Offt2-2	GGAAAAGCGCGATGAGGCAG
Klf5 Offt3-1	GCGAAAATGCGTCTCGCTGC
Klf5 Offt3-2	TCGGAGAGACGATAGGGCTCTG
Npr3 Offt1-1	GCAGCTGATTCTAGCCATAATTACA
Npr3 Offt1-2	GGCTGACCTGGAACTCACTC
Npr3 Offt2-1	CTTTGGTGGGCTTAGGTGCAGT
Npr3 Offt2-2	CCCTCCTCCTTTTGACTCTTAGC
Npr3 Offt3-1	CGGAAGTACAGATCTGAAGTGTGA
Npr3 Offt3-2	CAGCTGTGACGTGCTACAGAGTAA

Supplementary Information (the full-length gel data)

Figure 3 (c)

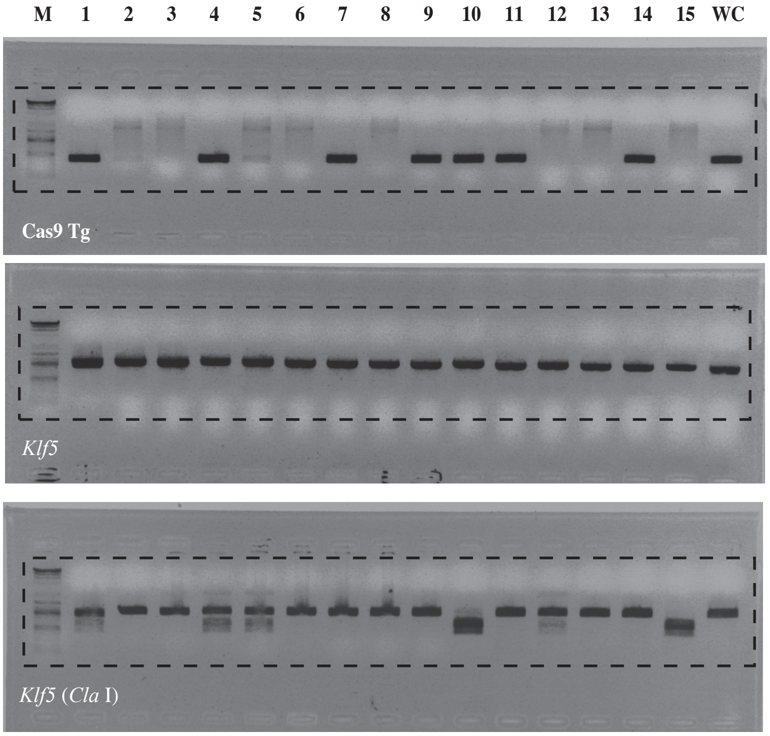

Figure S2 (a)

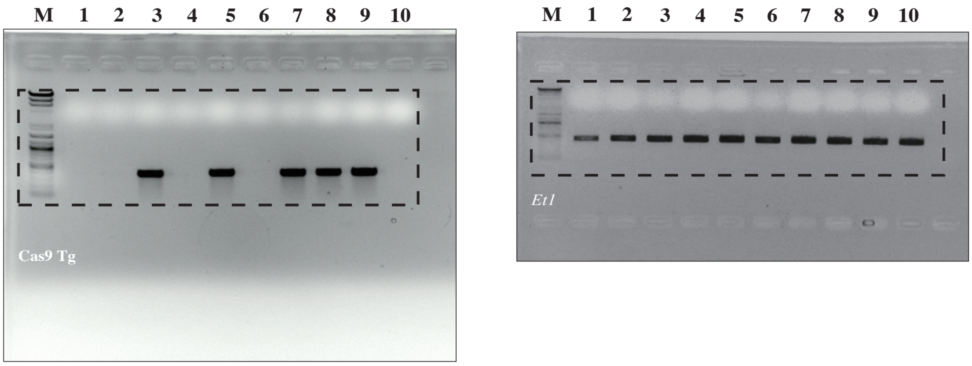

Supplementary Information (the full-length gel data)

Figure S7

(c)

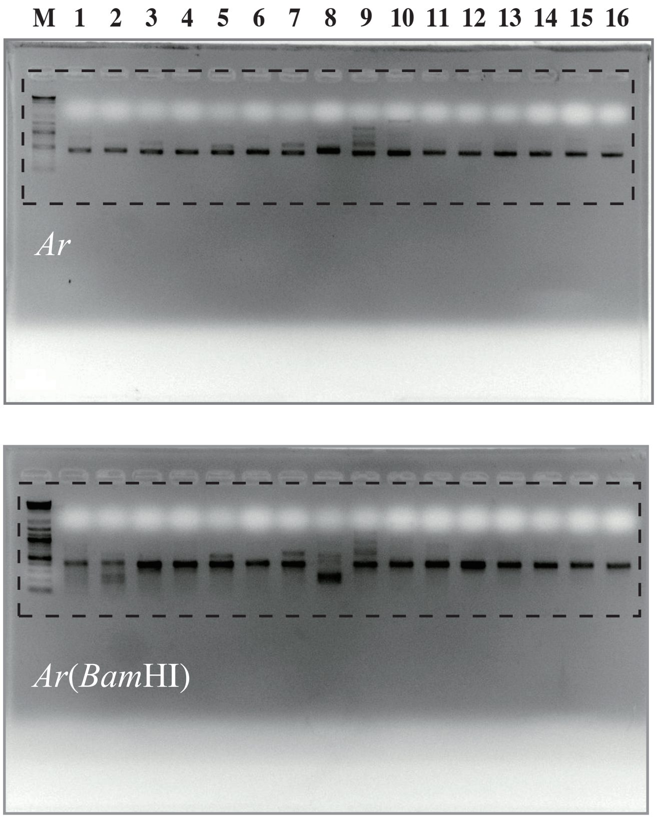

Supplementary Information (the full-length gel data)

Figure S9

(a)

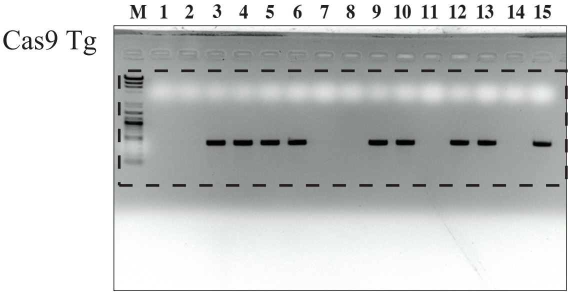

(b)

Et1

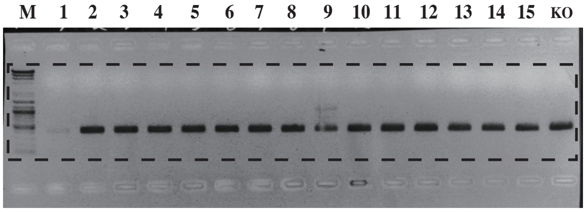

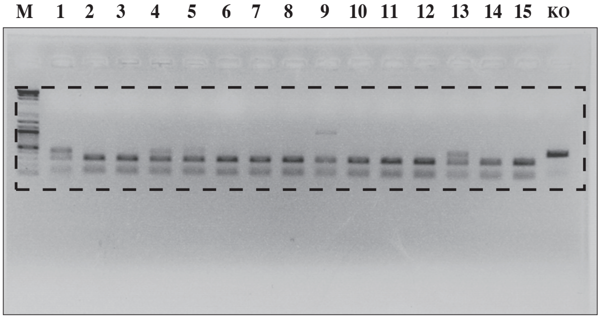

Ramp 1

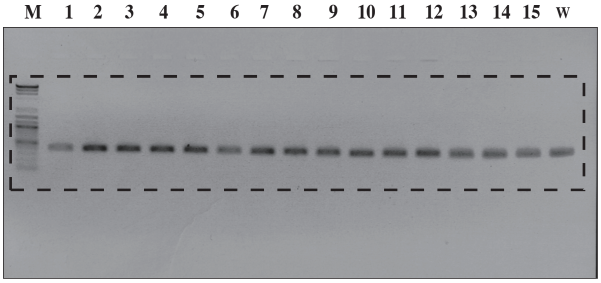

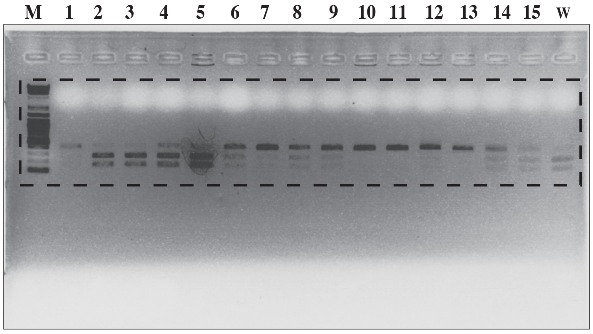

Ramp 3

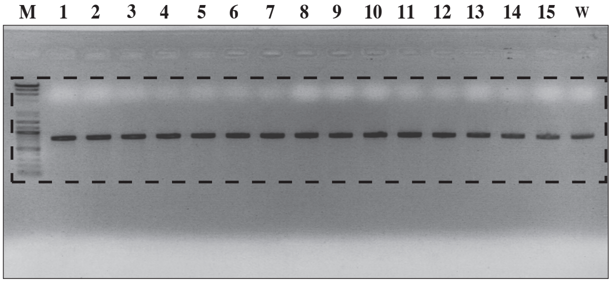

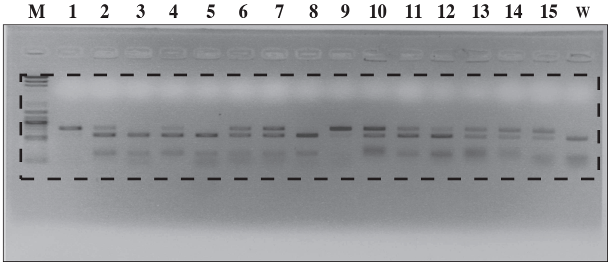

Supplement: Supplementary file 1 — Dataset1. [file 41598_2020_57996_MOESM1_ESM.pdf]
